# Integrative prediction of Alzheimer's disease and related dementias using multi‐omics aging clocks and genetic data

**DOI:** 10.1002/alz.71869

**Published:** 2026-09-28

**Authors:** Shayan Mostafaei, Karolina Gustavsson, Hampus Hagelin, Jonathan K. L. Mak, Trung Nghia Vu, Ida K. Karlsson, Sara Hägg

**Affiliations:** ^1^ Department of Medical Epidemiology and Biostatistics Karolinska Institutet Stockholm Sweden; ^2^ Department of Pharmacology and Pharmacy, Li Ka Shing Faculty of Medicine The University of Hong Kong Hong Kong China

**Keywords:** Alzheimer's disease, biological aging, dementia, metabolomics, polygenic risk score, proteomics

## Abstract

**INTRODUCTION:**

We tested whether combining Alzheimer's disease and related dementias (ADRD) polygenic risk scores (PRS) with biological aging markers improves ADRD risk prediction.

**METHODS:**

In 16,215 UK Biobank (UKB) participants free of ADRD at baseline (median follow‐up: 10.08 years), 397 incident diagnoses were identified. Biological aging measures included clinical, telomere, proteomic, and metabolomic aging markers. External validation/replication was performed in TwinGene (*N* = 3772; 331 cases).

**RESULTS:**

The fully integrated model achieved area under the curve (AUC) = 0.90 and area under the precision‐recall curve (AUPRC) = 0.24 in the UKB held‐out test set. PRS was the strongest predictor (subdistribution hazard ratio [sHR] = 2.24, 95% CI: 2.02–2.48), followed by ProtAge (sHR = 2.01, 95% CI: 1.13–3.58). The top predicted‐risk quartile showed higher ADRD incidence (sHR = 16.73, 95% CI: 6.49–43.11). TwinGene showed moderate transportability (AUC = 0.757; AUPRC = 0.223) and preserved risk stratification (sHR = 4.02, 95% CI: 3.25–5.02).

**DISCUSSION:**

Integrating PRS and biological aging measures improved ADRD risk stratification.

## Highlights

- PRS and biological aging markers improved Alzheimer's disease and related dementias (ADRD) risk stratification.
- The integrated model achieved area under the curve (AUC) = 0.90 and area under the precision‐recall curve (AUPRC) = 0.24 in the UK Biobank (UKB).
- Polygenic risk score (PRS) and ProtAge were leading contributors to predicted ADRD risk.
- TwinGene validation showed moderate transportability and risk stratification.

## 1 BACKGROUND

Morbidity and mortality increase significantly with chronological age (CA); however, there is heterogeneity in the aging process. This heterogeneity can be captured by the concept of biological aging (BA), and reflects functional decline across different systemic levels, from molecules all the way to organs and physiological systems.^1^ Different aging clocks, including DNA methylation–based epigenetic clocks, proteomic clocks, metabolomic clocks, and clinical/composite indices, have been developed to capture different aspects of aging^2^, ^3^ and differ in their ability to predict disease risk.^4^, ^5^ These clocks yield scores that can be interpreted relative to CA, with accelerated BA during mid‐life linked to increased risk of Alzheimer's disease and related dementias (ADRD),^5^, ^6^ a syndrome which has an increased incidence in late life.^7^

ADRD is a syndrome characterized by impaired neurological function, with Alzheimer's disease (AD) and vascular dementia (VaD) among the most common subtypes.^8^ Hallmarks of aging and neurodegeneration overlap, such as impaired proteostasis, mitochondrial dysfunction, and chronic inflammation.^1^, ^9^, ^10^ Both genetic^11^ and environmental factors^12^ contribute to ADRD risk. While rare familial forms show high penetrance, most cases are sporadic with polygenic contributions revealed through genome‐wide association studies (GWAS). There are over 80 genetic loci associated with ADRD, with the apolipoprotein E (*APOE*) ε4 allele as the strongest risk factor.^11^, ^13^

At the molecular level, alongside early *β*‐amyloid and tau pathology, proteomic alterations can be detected more than a decade before clinical diagnosis, with plasma proteins including glial fibrillary acidic protein (GFAP), neurofilament light chain (NEFL), and growth differentiation factor 15 (GDF15) reflecting astroglial activation, neuroaxonal injury, and stress‐response pathways implicated in ADRD pathophysiology.^14^ Metabolomic changes highlight disruptions in lipid and energy metabolism in ADRD, where lipid metabolites such as low‐density lipoprotein (LDL) and high‐density lipoprotein (HDL) play critical roles in maintaining neuronal function and blood–brain barrier integrity.^15^

**RESEARCH IN CONTEXT**

1. **Systematic review**: We reviewed studies of Alzheimer's disease and related dementias (ADRD) risk prediction, polygenic risk scores (PRS), biological aging measures, and omics‐based aging clocks. Prior work supports associations of genetic risk, clinical biological aging, proteomic markers, and metabolomic profiles with dementia risk, but few studies have integrated these domains within a unified prediction framework with calibration, decision‐curve analysis, competing‐risk risk stratification, and external validation.
2. **Interpretation**: In UK Biobank, integrating ADRD PRS with clinical, proteomic, and metabolomic aging measures improved risk stratification beyond demographic, lifestyle, and genetic predictors alone. External validation/replication in TwinGene showed moderate transportability and preserved risk stratification, while also highlighting expected performance attenuation across cohorts and omics‐platform settings.
3. **Future directions**: Further validation is needed in diverse ancestry groups, independent cohorts, and different omics platforms. Future work should evaluate prospective calibration, cost‐effectiveness, decision thresholds, repeated omics measurements, and biomarker‐defined ADRD subtypes before clinical implementation.

Recent advances in multi‐omics have enabled a systems‐level view of biological aging and neurodegeneration by integrating genomic, proteomic, metabolomic, and clinical biomarker information. Prior work, including emerging preprint evidence, suggests that omics‐based aging clocks and multi‐layer molecular profiling can capture complementary disease‐relevant processes and improve risk prediction beyond demographic or genetic factors alone.^16^, ^17^, ^18^, ^19^ However, AD pathology markers alone do not fully capture systemic aging‐related processes or mixed‐pathology risk that contribute to broader ADRD. Complementary blood markers can reflect astroglial activation and neuroaxonal injury, but they are less specific to AD pathology.^14^, ^20^ These considerations underscore the need for integrated, systems‐level approaches, combining genetic risk with multi‐domain BA measures, to improve risk stratification and prevention across ADRD.

Building on this evidence, combining ADRD polygenic risk with multi‐domain biological aging measures may improve risk stratification by integrating inherited susceptibility with systemic physiological dysregulation.^11^, ^16^, ^21^ Because ADRD prevention and trial enrichment increasingly require identification of higher‐risk individuals before clinical impairment, robust predictive frameworks are needed.^22^, ^23^, ^24^ In this study, we tested whether integrating ADRD PRS with clinical, proteomic, and metabolomic biological aging measures improves prediction and risk stratification for incident ADRD in UK Biobank (UKB), with external validation in TwinGene.

## 2 METHODS

### 2.1 Study design and population

The UKB is a population‐based longitudinal cohort of ∼500,000 individuals aged 37 to 73 years recruited across the United Kingdom between 2006 and 2010. Our analytic sample included 16,215 participants free of ADRD at baseline with available PRS and biological aging measures (median follow‐up: 10.08 years). Individuals with missing values for any biological markers across various omics levels were excluded from the analysis. Participants who requested to withdraw from the study, as indicated in the most recent list of withdrawals in the UKB, and those with genetically non‐European ancestry or close relatives were also excluded. To address population heterogeneity, all models were adjusted for baseline age, sex, body mass index (BMI), smoking status, alcohol intake frequency, education level, and the top 10 genetic principal components. Sensitivity analyses were conducted across subgroups defined by sex, age‐at‐onset, and *APOE* status to evaluate robustness of findings. An overview of participant selection and the analytic workflow is shown in Figure 1. To evaluate potential selection bias due to the availability of omics or biomarker data, we compared main characteristics between included participants and excluded participants from the eligible UKB sample using standardized mean differences (SMD).

The UKB study was approved by the North West Multi‐Centre Research Ethics Committee and the specific research aim was further approved by the Swedish Ethical Review Authority (Dnr. 2022‐06634‐01). All participants provided written informed consent. Analyses used fully de‐identified data and adhered to the Declaration of Helsinki. This study followed the Transparent Reporting of a multivariable prediction model for Individual Prognosis or Diagnosis (TRIPOD) reporting guideline for prediction model development and validation and the Strengthening the Reporting of Observational Studies in Epidemiology (STROBE) reporting guideline for observational cohort studies.^25^

### 2.2 Genetic data

This study leveraged UKB genome‐wide genotype data from the v3 genotyping release (488,377 participants), generated using two closely related custom arrays (the UK Biobank Axiom Array and the UK BiLEVE Axiom Array; Affymetrix/Applied Biosystems) and imputed using the 1000 Genomes Project and Haplotype Reference Consortium panels to approximately ∼93 million variants.^26^, ^27^ Participants were excluded according to UKB sample‐level quality control (QC), including withdrawal from the study, discordance between self‐reported and genetically inferred sex and/or sex chromosome aneuploidy, unusually high heterozygosity, and elevated genotype missingness (e.g., call rate < 95%).^27^ We then applied additional variant‐level filters, excluding rare variants (minor allele frequency < 1%), variants with imputation INFO score < 0.6, and variants deviating from Hardy–Weinberg equilibrium (*p* < 1 × 10^−^^12^). Related individuals (Data‐Field 22021) were excluded to retain an unrelated analytic set. After applying these QC steps and ancestry filtering, 276,566 unrelated individuals of European ancestry were retained for polygenic risk score (PRS) modeling, comprising ∼9 million variants after QC.

### 2.3 PRS

We defined the PRS variant panel using genome‐wide significant lead variants reported by Bellenguez et al. (2022), which identified 83 SNPs across 75 loci from over 111,000 participants with ADRD and 677,000 participants without ADRD.^11^ Because the Bellenguez GWAS included UKB participants, we restricted the PRS to 80 linkage disequilibrium (LD) ‐independent single nucleotide polymorphisms (SNPs) (including the *APOE* locus) that were available and harmonizable in the UKB‐excluded Jansen phase‐1 summary statistics with effect size estimates.^28^ *APOE* genotype was not entered separately as an independent predictor in the primary prediction models; *APOE* information was incorporated through the PRS including the *APOE* locus. *APOE‐ε4* carrier status was used only for stratified sensitivity analyses. Jansen phase‐1 effect sizes were used to weight these SNPs for PRS construction. We applied a deep data‐driven machine learning (DDML) approach using a Bayesian variational autoencoder trained on individual‐level genotype data from the 276,566 unrelated individuals of European ancestry in the UKB.^21^ The architecture included a three‐layer encoder (512 → 256 → 128 units) and a symmetric decoder, with a 50‐dimensional latent space defined via the reparameterization trick. SNP effect sizes and standard errors informed the priors for latent variables, integrated into the Kullback–Leibler divergence term of the loss function. The model was trained for 100 epochs using the Adam optimizer (learning rate = 0.001, batch size = 256), with early stopping based on evidence lower bound objective (ELBO). The final PRS was derived from the posterior mean of the latent variables.^21^ Implementation details are provided in the Code Availability statement.

### 2.4 BA measures

To derive biological age estimates, we analyzed 325,870 UKB participants with complete data for 18 biomarkers, including HbA1c and total cholesterol, as utilized in BA algorithms.^4^ The 18 biomarkers were selected based on prior validation and their correlation with CA (|r|> 0.1). Biological age was estimated using three composite measures derived from blood chemistry and clinical data: the Klemera–Doubal method (KDM), PhenoAge, and homeostatic dysregulation (HD).^29^, ^30^, ^31^ Details on the calculation and interpretation of these measures have been described previously.^32^ These measures were computed using “BioAge” R package.^33^ In addition to these BA measures, we calculated the frailty index (FI) according to the Rockwood deficit accumulation model,^34^ as validated in the UKB^35^ and used relative leukocyte telomere length (TL) adjusted for the influence of technical parameters in the UKB.^36^

### 2.5 Metabolomic aging clock

UKB Nightingale NMR metabolomics data were generated from ethylenediaminetetraacetic acid (EDTA) plasma using a high‐throughput NMR metabolomics platform (Nightingale Health Ltd., Helsinki, Finland), providing 251 metabolic biomarkers released in multiple phases. For MetaboAge development, we used the UKB NMR metabolomics release available at the time of analysis (Phase 2), retaining 228,513 participants after QC. To construct the metabolomic BA clock (MetaboAge), we utilized 184 curated NMR‐based metabolic features selected to minimize redundancy. MetaboAge was trained to predict CA. Raw metabolic measurements were normalized using a Box–Cox transformation to handle non‐negative, skewed distributions, and missing values were imputed using k‐nearest neighbors (*k* = 9). Outlier detection was performed using Mahalanobis distance computed from a robust covariance estimator to improve stability in the presence of correlated, high‐dimensional features. The clock was developed using a stacked ensemble model that combined XGBoost, LightGBM, and CatBoost base learners, with an Elastic‐Net (alpha = 0.5) model serving as the meta‐learner to predict CA.^37^, ^38^ Separate models were trained for men and women to account for sex‐specific differences. To avoid information leakage in downstream ADRD prediction, the ADRD analytic cohort was first split into training (70%) and test (30%) sets. MetaboAge was then trained only within the training set using five‐fold cross‐validation (internal cross‐fitting) to generate out‐of‐fold MetaboAge estimates for training participants. The finalized MetaboAge model (fit on the full training set) was subsequently used to generate MetaboAge predictions for participants in the held‐out test set. The clock estimation followed our previously described pipeline; implementation details are provided in the Code Availability statement.

### 2.6 Proteomic aging clock

For the proteomic BA clock (ProtAge), we analyzed normalized protein expression (NPX; log2 scale) data for 2937 protein assays (all available assays after QC; no protein‐level feature selection) from 50,482 UKB participants, measured using the Olink platform. ProtAge was trained to predict CA. As with the metabolomic data, missing values were imputed using a k‐nearest neighbors (*k* = 9) approach. Outliers were identified and excluded using the Isolation Forest (iForest) algorithm. The BA clock was built using a stacked ensemble model that combined XGBoost, LightGBM, and CatBoost as base learners, with an Elastic‐Net (alpha = 0.5) model serving as the meta‐learner. Separate models were trained for men and women to account for sex‐specific differences.^39^ To avoid information leakage in downstream ADRD prediction, the ADRD analytic cohort was first split into training (70%) and test (30%) sets. ProtAge was trained only within the training set using five‐fold cross‐validation to obtain out‐of‐fold ProtAge estimates for training participants. The finalized ProtAge model (fit on the full training set) was then used to generate ProtAge predictions for the held‐out test set. Implementation details are provided in the Code Availability statement.

### 2.7 ADRD

Incident ADRD diagnoses were ascertained based on the International Classification of Diseases, 10th Revision (ICD‐10) codes obtained from hospital inpatient and death registry records, validated in prior UKB studies.^40^ This included codes for ADRD (F00, F01, F02, F03, G30, G31.0, G31.1, G31.8).^40^, ^41^ We analyzed ADRD as a broad composite endpoint to maximize event capture and reflect registry‐based dementia ascertainment; subtype categories were summarized descriptively, but subtype‐specific multi‐omics prediction models were not fitted because of limited case numbers within individual dementia subtypes and potential diagnostic overlap in registry‐coded phenotypes. Follow‐up time was defined from baseline to the earliest of ADRD diagnosis, death, or censoring (end of follow‐up in March 2023). Participants with a diagnosis or a self‐reported history of neurological disorders before baseline were excluded from the analysis.^6^

### 2.8 Overview of analytic workflow

We used a split‐sample framework to prevent information leakage and to link each modeling component transparently. (1) We first defined the analytic cohort and performed a stratified 70/30 training–test split by incident ADRD status. (2) Within the training split only, we derived omics‐based aging clocks (ProtAge and MetaboAge) using cross‐fitting; the resulting clock predictions were then computed for the held‐out test split using the trained clock models. (3) We trained stepwise XGBoost classifiers in the training split and evaluated all models once on the held‐out test split, reporting discrimination (ROC‐AUC and AUPRC) and calibration. (4) Using the final integrated predictor set (Model 5), we fit Fine–Gray competing‐risk regression with death as a competing event and estimated absolute ADRD risk at prespecified horizons. (5) Finally, we assessed clinical interpretability via risk stratification (e.g., top 25% vs. bottom 75% predicted risk) and cumulative incidence curves in the held‐out test split and in external validation (TwinGene). All analyses were designed for prediction, risk stratification, and association assessment; they should not be interpreted as estimating causal effects of biological aging measures, PRS, or omics‐derived clocks on ADRD risk.

### 2.9 Predictive models

We partitioned the ADRD analytic cohort into training (70%; *n* = 11,351, including 275 incident ADRD cases and 11,076 non‐cases) and held‐out test (30%; *n* = 4864, including 122 incident ADRD cases and 4742 non‐cases) sets using a reproducible, stratified random split by ADRD status, sex, and baseline age bin (5‐year bins), with a fixed seed. Model development, including hyperparameter tuning, was performed using five‐fold cross‐validation within the training set, and the held‐out test set was used only for final performance evaluation to minimize optimism and information leakage. Omics clocks (ProtAge and MetaboAge) were then trained within the training set and applied to the test set to generate clock predictors used in ADRD modeling. To develop multivariable models, we integrated PRS, BA measures (PhenoAge, FI, TL), and omics‐based aging clocks (ProtAge and MetaboAge) using an XGBoost‐based ensemble classification algorithm. KDM and HD were evaluated in univariate and correlation analyses; for multivariable prediction we prioritized PhenoAge, FI, and TL to reduce collinearity and retain clinically interpretable aging domains, while preserving coverage of complementary aging constructs. The final model combined all predictors and was evaluated for discrimination, calibration, and competing‐risk risk stratification. We trained XGBoost classifiers for discrimination (area under the receiver operating characteristic curve [AUC]) and then fit Fine–Gray models to estimate subdistribution hazard ratio (sHR) and cumulative incidence accounting for death.^42^, ^43^ The stepwise XGBoost models were used to quantify incremental discrimination (how well successive predictor sets distinguish individuals who later develop ADRD from those who do not), whereas the Fine–Gray models—fit using the final integrated predictor set (Model 5)—were used to estimate absolute ADRD risk over time while accounting for death as a competing event. Because these approaches target different estimands (prediction vs. adjusted association under competing risks), they are not expected to yield identical “importance”/significance patterns for individual predictors. All models were adjusted for baseline age, sex, BMI, alcohol intake frequency, smoking status, education level, and the top 10 genetic principal components. Predictors were added stepwise, including PRS (including the *APOE* locus, without separate *APOE* genotype entry), BA measures (PhenoAge, FI, TL), ProtAge, and MetaboAge.

### 2.10 Survival analysis and composite risk stratification

To assess the prognostic value of the final multivariable model for predicting incident ADRD, we constructed a composite risk score using the final and most comprehensive model, including CA, PRS, PhenoAge, FI, TL, ProtAge, MetaboAge, and covariates. Follow‐up time was defined from baseline to ADRD diagnosis, death, or censoring; competing events were coded as ADRD (event = 1), death (event = 2), or censoring (event = 0). Fine–Gray competing risk regression was used to model the subdistribution hazard, and absolute risks were predicted at 5‐ and 9‐year horizons to reflect clinically relevant mid‐term prediction and the median follow‐up duration of the cohort, ensuring robust estimates across short‐ and long‐term intervals. Individuals were stratified into high‐risk (top 25%) and low‐risk (bottom 75%) groups based on predicted risk quantiles, and subdistribution hazard ratio (sHR) was estimated to quantify differences in risk between groups. We used time since baseline assessment (days) as the time scale in Cox and Fine–Gray models; CA was included as a covariate.

#### 2.10.1 External validation and replication in TwinGene

We used TwinGene, a Swedish Twin Registry–based population cohort, as an independent cohort to evaluate external transportability of the integrated ADRD prediction framework.^44^ The complete‐case TwinGene downstream cohort included 3772 participants, including 331 incident ADRD cases and 841 competing death events. Predictors were harmonized to mirror the UKB Model 5 structure where possible. The ADRD PRS used the same selected SNP panel and external Jansen phase‐1 GWAS summary‐statistic weights used in UKB, with 73 SNPs available and harmonizable in TwinGene.^11^, ^21^, ^28^ Clinical biological aging measures followed harmonized PhenoAge, frailty‐index, and telomere‐length frameworks.^30^, ^45^ Because omics platforms and feature availability differed between cohorts, TwinGene analyses used a cohort‐trained Olink ProtAge replication and a UKB‐portable recalibrated MetaboAge variable; thus, TwinGene was interpreted as external transportability and methodological replication rather than strict frozen‐coefficient validation of every omics component.^37^, ^38^, ^39^

### 2.11 Statistical analysis

All preprocessing and model fitting (including omics‐clock derivation) were conducted in the training set and evaluated in the held‐out test set to minimize information leakage. Categorical predictors were encoded using model‐matrix/dummy‐variable encoding before fitting XGBoost. The encoding scheme was derived in the training set and then applied to the held‐out test set, with feature columns aligned between training and test matrices to ensure consistent feature representation and avoid information leakage. Predictive performance was primarily assessed using the area under the ROC curve (AUC) in the held‐out test set, with model comparisons based on pairwise AUC tests. Given the class imbalance, we additionally evaluated AUPRC in the held‐out test set, with 95% confidence intervals estimated using stratified nonparametric bootstrap resampling by ADRD status. To assess robustness to split variability in this low‐incidence setting, we repeated the stratified 70/30 train–test split and full modeling pipeline 100 times (stratified by ADRD status, sex, and baseline age bin [5‐year bins]) and summarize the empirical mean and 2.5th–97.5th percentiles for AUC/AUPRC, alongside a logistic‐regression benchmark. Calibration was assessed in the held‐out test set both visually (decile‐based calibration plots) and quantitatively using logistic recalibration metrics (calibration intercept and slope) and the Brier score. Decision‐curve analysis was performed in the held‐out test set to evaluate the net benefit of the final integrated model across low risk‐threshold ranges relevant to ADRD risk enrichment. In TwinGene, external validation/replication performance was evaluated using ROC‐AUC, AUPRC, and competing‐risk risk stratification with harmonized available predictors. For the fully integrated model, we used SHapley Additive exPlanations (SHAP) to quantify each predictor's contribution to individual‐level risk predictions and to summarize global feature importance. SHAP values were computed on the held‐out test set, and results are presented as SHAP summary plots. We prespecified a set of sensitivity analyses to test robustness of discrimination and effect estimates across subgroups (sex, onset age, *APOE* strata), PRS specifications (with/without *APOE*), feature sets (with/without lifestyle), follow‐up horizons, missing‐data strategies (complete‐case vs. imputation), competing‐risk frameworks (Fine–Gray vs. cause‐specific Cox), and alternative survival time scales (time since baseline vs. attained age). Medication use, hypertension, diabetes, and cardiovascular disease were not modeled as separate sensitivity analyses. Detailed analytic steps for all sensitivity analyses, including time‐scale definitions and censoring rules, are provided in Supplementary Methods A.M1. TwinGene validation and harmonization details are provided in Supplementary Methods A.M2. All analyses were performed using a combination of R version 4.4.3 and Python 3.10, integrating statistical modeling, machine learning, and visualization workflows. In R, we used the packages “survival”, “riskRegression”, “pROC”, “cmprsk”, “prodlim”, “survminer”, and “ggplot2” for ROC analysis, competing risk modeling, and cumulative incidence visualization.^46^, ^47^, ^48^, ^49^ BA measures including PhenoAge, KDM, and HD were computed using the “BioAge” package.^33^ The MetaboAge and ProtAge clocks were constructed in R using a stacked ensemble framework combining gradient‐boosting learners (“xgboost”, “lightgbm”, “catboost”; implemented via “caretEnsemble”), with Elastic‐Net regression (“glmnet”) as the meta‐learner.^50^, ^51^, ^52^ Hyperparameters were optimized within the training split using cross‐validation with a fixed seed, and full tuning specifications and package versions are provided in the GitHub repository. Preprocessing steps included Box‐Cox transformation (via “car” and “forecast” packages), K‐Nearest Neighbors (KNN) imputation using “VIM”, and outlier detection via Mahalanobis distance (“stats”) and Isolation Forest (“isotree”).^53^, ^54^ For PRS modeling, we implemented a Bayesian variational autoencoder in Python using “TensorFlow”, “Keras”, and “scikit‐learn” libraries.^55^ We controlled the false discovery rate (FDR) using the Benjamini–Hochberg procedure; FDR values (q‐values) are reported.^56^ Implementation details are provided in the Code Availability statement.

## 3 RESULTS

### 3.1 Study population

From the initial UKB cohort of approximately 500,000 participants, we applied strict inclusion criteria to ensure complete data across genetic, proteomic, metabolomic, and clinical biomarkers. After excluding individuals with prior neurological diagnoses, non‐European ancestry, relatedness, and missing data, the final analytic sample comprised 16,215 ADRD‐free participants at baseline. The mean age of participants was 56.98 years (SD = 7.88), and 45.8% were men. During a median follow‐up of 10.08 years (interquartile range: 8.98–10.9 years), 397 (2.45%) incident ADRD diagnoses were reported, including Alzheimer's disease (49%), vascular or mixed dementia (19%), and other or unspecified dementia (32%). The mean age at diagnosis was 74.43 years (SD = 5.47), and the cumulative incidence of all‐cause mortality during follow‐up was 9.3%. Covariates including smoking, alcohol intake frequency, education level, and BMI are also summarized in Table 1. Main characteristics were similar between included and excluded participants (all standardized mean differences < 0.10; Table S1).

**TABLE 1:** Characteristics of included UK Biobank participants.

	All participants	ADRD	Non‐ADRD
Characteristics	(*N* = 16,215)	(*N* = 397)	(*N* = 15,818)
Follow‐up time, years (median [IQR])	10.08 [8.98–10.90]	8.89 [7.39–10.17]	10.12 [9.01–10.90]
Age, years (mean ± SD)	56.98 ± 7.88	64.67 ± 4.45	56.78 ± 7.84
Age at ADRD onset, years (mean ± SD)	NA	74.43 ± 5.47	NA
*APOE‐ε4* carriers (*N*, %)	2423 (15%)	159 (40%)	2264 (14.3%)
Sex, men (*N*, %)	7422 (45.8%)	200 (50%)	7222 (45.7%)
Deaths during follow‐up (*N*, %)	1510 (9.3%)	212 (53.4%)	1298 (8.2%)
ADRD PRS (mean ± SD)	−0.59 ± 1.30	0.92 ± 1.61	−0.63 ± 1.27
PhenoAge, years (mean ± SD)	50.88 ± 9.26	59.45 ± 7.14	50.66 ± 9.20
Klemera–Doubal Method, years (mean ± SD)	55.76 ± 8.74	63.40 ± 5.72	55.64 ± 8.72
Homeostatic dysregulation (mean ± SD)	2.18 ± 0.87	2.42 ± 1.01	2.17 ± 0.87
Frailty index (median [IQR])	0.11 [0.07–0.16]	0.14 [0.10–0.20]	0.11 [0.07–0.16]
Telomere length (mean ± SD)	0.83 ± 0.14	0.79 ± 0.12	0.83 ± 0.14
ProtAge, years (mean ± SD)	56.97 ± 7.75	64.48 ± 4.24	56.78 ± 7.73
MetaboAge, years (mean ± SD)	56.94 ± 5.85	61.38 ± 4.39	56.83 ± 5.84
Body mass index, kg/m^2^ (mean ± SD)	27.32 ± 4.52	27.32 ± 4.76	27.32 ± 4.51
Alcohol intake frequency, ordinal categories (mean ± SD)	2.88 ± 1.53	3.20 ± 1.69	2.87 ± 1.53
Smokers (current or former, *N*, %)	7253 (44.7%)	179 (45.1%)	7074 (44.7%)
Education level, high or intermediate (*N*, %)	13253 (81.7%)	263 (66.2%)	12990 (82.1%)

*Note*: Values are presented as mean ± standard deviation (SD), median [interquartile range (IQR)], or number (percentage), as appropriate. Continuous variables are summarized as mean ± SD when approximately normally distributed and as median [IQR] when skewed. ADRD, Alzheimer's disease and related dementias. NA, Not available. Apolipoprotein E (*APOE) ‐ε4* carriers: Number and percentage of participants carrying at least one *APOE‐ε4* allele. High or intermediate education is defined as ≥10 years of formal education. Smoking status includes both current and former smokers. Alcohol intake frequency is UK Biobank Field 1558 (ordinal categories 1–7: daily or almost daily to never); values are shown as mean ± SD. Biological aging measures include Phenotypic Age (PhenoAge), Klemera–Doubal Method (KDM), homeostatic dysregulation (HD), Frailty Index (FI), and adjusted telomere length (TL; UK Biobank leukocyte telomere length adjusted for technical/assay parameters). FI was calculated as a 49‐item Rockwood frailty index (range from 0 to 1), representing the proportion of deficits present. Polygenic risk scores (PRS) for ADRD are calculated using Bayesian variational autoencoder method including the *APOE* region. PRS values are shown on the original scale used in the model (higher values indicate higher genetic risk).

### 3.2 Univariate analyses

PRS scores were higher in participants with ADRD compared to participants without ADRD (mean 0.92 vs. ‐0.63; *p* < 1×10^−^^4^; Figure S1). To evaluate the accuracy of omics‐based BA clocks, we trained ProtAge and MetaboAge models on stratified sex subgroups. The scatter plots revealed strong correlations with CA: ProtAge achieved a Pearson correlation of *r* = 0.97 and root mean square error (RMSE) = 1.95, while MetaboAge reached *r* = 0.74 and RMSE = 5.28 (Figures S2 and S3). When comparing ADRD group to non‐ADRD group, individuals with ADRD exhibited accelerated BA across all clocks. Median values were higher in ADRD group for ProtAge (64.48 vs. 56.78 years), MetaboAge (61.38 vs. 56.83 years), PhenoAge (59.45 vs. 50.66 years), and KDM (63.40 vs. 55.64 years). HD (2.42 vs. 2.17), FI (0.14 vs. 0.11), and TL (0.79 vs. 0.83) similarly showed statistically significant differences between groups, with ADRD group consistently reflecting greater molecular and physiological aging or dysregulation.

ProtAge and CA showed the highest univariate discriminative ability for predicting ADRD, each with an AUC of 0.81 (95% CI: 0.79–0.83), followed by PhenoAge (AUC = 0.78, 95% CI: 0.75–0.79) and KDM (AUC = 0.77, 95% CI: 0.75–0.79). MetaboAge showed an AUC of 0.73 (95% CI: 0.71–0.75), whereas PRS showed an AUC of 0.70 (95% CI: 0.68–0.72). Other BA measures exhibited modest predictive ability: FI (AUC = 0.62, 95% CI: 0.59–0.65), HD (AUC = 0.58, 95% CI: 0.55–0.61), and TL (AUC = 0.57, 95% CI: 0.55–0.60). Principal component analysis (PCA) of environmental/lifestyle factors (smoking, alcohol intake frequency, BMI, and education) yielded a first principal component (PC1) explaining 24% of the variance and showing limited discrimination for ADRD (AUC = 0.57, 95% CI: 0.545–0.605). Therefore, we did not use a PCA‐derived composite environmental score (PC1) as a predictor; instead, lifestyle factors were retained as individual covariates in the multivariable models. These univariate AUC estimates are summarized in the forest plot (Figure 2).

### 3.3 Associations among BA measures, PRS, and lifestyle covariates

We assessed associations of lifestyle and demographic factors (BMI, smoking, alcohol intake frequency, education, and sex) with each biological aging measure and PRS using linear regression with FDR correction (Figure S4 and Table S2). Because predictors and outcomes are on different scales, Figure S4 summarizes results using standardized regression coefficients to facilitate comparison across covariates and measures, whereas Table S2 reports unstandardized *β* coefficients for interpretability. Overall, lower education showed the strongest and most consistent association with more advanced biological aging across multiple measures, particularly KDM, PhenoAge, ProtAge, MetaboAge, HD, and FI. Higher BMI was also consistently associated with advanced biological aging, with the largest standardized effects observed for HD and FI. PRS showed weak associations with lifestyle factors, with a statistically significant positive association observed only for low education (Figure S4 and Table S2). Correlations among biological aging measures indicated strong agreement among CA, ProtAge, PhenoAge, and KDM, whereas HD, FI, TL, and PRS were weakly correlated with most other measures (Figure 3).

### 3.4 Multivariable predictive modeling and risk stratification

To evaluate the additive value of genetic and BA predictors, we developed a series of multivariable predictive models and assessed discrimination for incident ADRD in a held‐out test set (*n* = 4864, including 122 incident ADRD cases). The base model including CA and sex yielded an AUC of 0.79 (95% CI: 0.76–0.82) and an AUPRC of 0.06 (95% CI: 0.05–0.09). Adding lifestyle factors (smoking, alcohol intake frequency, BMI, and education) improved discrimination (Model 1: AUC = 0.82, *p* = 0.035; AUPRC = 0.09 (95% CI: 0.06–0.14)). Incorporating the PRS further increased performance (Model 2: AUC = 0.86, *p* < 0.0001; AUPRC = 0.17 (95% CI: 0.12–0.26)), highlighting the contribution of genetic risk. Adding BA measures (PhenoAge, FI, and TL) yielded additional gains (Model 3: AUC = 0.89, *p* = 0.002 vs. Model 2; AUPRC = 0.19 [95% CI: 0.13–0.28]). ProtAge provided only a marginal increment (Model 4: AUC = 0.90; AUPRC = 0.22 (95% CI: 0.15–0.32)), with no statistically significant improvement over Model 3 (*p* = 0.714; AUC comparison). Adding MetaboAge did not further improve discrimination (Model 5: AUC = 0.90; *p* = 0.380) but increased AUPRC to 0.24 (95% CI: 0.16–0.34).

Given class imbalance, we report AUPRC for all stepwise models (Table 2 and Figure S5), which increased progressively across model steps (0.06 → 0.24), indicating improved precision‐recall performance even when AUC gains plateaued. Accordingly, we interpret model performance primarily using AUPRC/precision–recall curves (Figure S5) and calibration (Figure S6).

**TABLE 2:** Stepwise comparison of multivariable models for predicting incident Alzheimer's disease and related dementias in the UK Biobank cohort.

Model	AUPRC (95% CI)	AUC (95% CI)	*p*‐value (vs. Base)	*p*‐value (vs. Model 1)	*p*‐value (vs. Model 2)	*p*‐value (vs. Model 3)
Base model: Age + Sex	0.06 (0.05–0.09)	0.79 (0.76–0.82)	Ref.	—	—	—
Model 1: Base model + Lifestyle	0.09 (0.06–0.14)	0.82 (0.79–0.84)	0.035	Ref.	—	—
Model 2: Model 1 + PRS	0.17 (0.12–0.26)	0.86 (0.83–0.89)	<0.0001	<0.0001	Ref.	—
Model 3: Model 2 + PhenoAge + FI + TL	0.19 (0.13–0.28)	0.89 (0.86–0.92)	<0.0001	<0.0001	0.002	Ref.
Model 4: Model 3 + ProtAge	0.22 (0.15–0.32)	0.90 (0.86–0.91)	<0.0001	<0.0001	0.001	0.714
Model 5: Model 4 + MetaboAge	0.24 (0.16–0.34)	0.90 (0.86–0.92)	<0.0001	<0.0001	<0.0001	0.380

*Note*: Area under the receiver operating characteristic curve (AUC) and area under the precision–recall curve (AUPRC) with 95% confidence intervals (CIs) are reported for each XGBoost model evaluated on the held‐out test set using a stratified 70/30 train–test split. Predictors include age, sex, polygenic risk score (PRS; including the apolipoprotein E [*APOE]* locus), biological aging measures (PhenoAge, frailty index, telomere length), and omics clocks (ProtAge, MetaboAge). Lifestyle factors include smoking, alcohol intake frequency, body mass index, and education. ProtAge and MetaboAge were generated using clock models trained only in the training split and applied to the test split. *P*‐values represent pairwise comparisons of AUCs on the same test set using DeLong's test; statistical significance was defined as *p*‐value < 0.05. As a benchmarking analysis using the same fixed train–test split and held‐out test set, logistic regression achieved an AUC of 0.79 (95% CI: 0.77–0.81) for the base model and an AUC of 0.86 (95% CI: 0.83–0.89) for the fully integrated predictor set corresponding to Model 5; repeated‐split logistic‐regression benchmarking is reported in Table S6.

At an illustrative default probability threshold of 0.5 (not optimized for this low‐incidence outcome), Model 5 yielded TP = 32, FP = 105, FN = 90, and TN = 4637, corresponding to specificity = 97.8%, sensitivity = 26.2%, positive predictive value (PPV) = 23.4%, F1 = 0.247, and accuracy = 96.0% in the held‐out test set. Because ADRD incidence is low, this default cutoff is provided only as an illustrative operating point; alternative, application‐driven thresholds (e.g., top 5% or 10% predicted risk for enrichment) are reported in Table S3 and show the expected precision–recall trade‐offs at lower risk thresholds.

*p*‐Values reflect pairwise comparisons of model AUCs on the same test set (Table 2). In a sensitivity analysis, we repeated the stepwise modeling using alternative predictor entry orders; the final model AUC was stable across orderings (AUC range 0.899‐0.904), indicating that overall discrimination was not driven by the order in which correlated predictors were added (Table S4). In addition to discrimination, the full model (Model 5) showed good agreement between predicted and observed risks in the test set, supported by calibration intercept/slope and Brier score (Figure S6). Decision‐curve analysis showed positive net benefit for the final integrated model across low predicted‐risk thresholds relevant to ADRD risk enrichment (Figure S7). We then used the final integrated predictor set (Model 5) to estimate absolute ADRD risk and risk stratification under competing mortality using Fine–Gray models. In parallel, competing‐risk modeling demonstrated clear separation of cumulative incidence between predicted risk strata, supporting the model's utility for risk enrichment.

The SHAP summary plot for Model 5 summarizes each predictor's contribution to the model's predicted ADRD risk (Figure S8). PRS had the largest mean absolute SHAP value, followed by CA, ProtAge, and PhenoAge. FI and TL also contributed, though with smaller effects. Lower BMI was associated with higher predicted risk, while alcohol intake frequency and smoking showed modest contributions. Low education and sex (men) had minimal influence compared with other predictors. MetaboAge contributed modestly and showed a model‐conditional pattern in which lower values tended to be associated with higher predicted risk, likely reflecting shared variance among correlated aging measures (Figure S8).

### 3.5 Survival and competing risks analysis

In the fully adjusted Fine–Gray competing‐risk model (Model 5) using time since baseline as the time scale, several predictors were associated with incident ADRD. PRS showed the strongest association (sHR per SD = 2.24, 95% CI: 2.02–2.48), followed by ProtAge (sHR = 2.01, 95% CI: 1.13–3.58). CA demonstrated a positive but non‐significant trend (sHR = 1.55, 95% CI: 0.88–2.73). Among other BA measures, FI was modestly associated with ADRD risk (sHR = 1.19, 95% CI: 1.07–1.33), while associations with PhenoAge (sHR = 1.08, 95% CI: 0.87–1.33), MetaboAge (sHR = 1.06, 95% CI: 0.88–1.28), and TL (sHR = 0.97, 95% CI: 0.87–1.09) were not statistically significant. The forest plot of sHRs of main predictors is shown in Figure 4. As a sensitivity analysis, we refit the Fine–Gray model using age as the time scale. The overall results were unchanged, with PRS remaining the strongest predictor and ProtAge remaining positively associated with ADRD risk, while the remaining BA measures showed no statistically significant associations.

Using the Fine–Gray model, we calculated each participant's 5‐year predicted risk of ADRD. Participants were stratified into high‐risk (top 25%) and low‐risk (bottom 75%) groups based on these predictions. The cumulative incidence of ADRD over time was substantially higher in the high‐risk group, as illustrated by the stratified curves. In a Fine–Gray regression comparing these groups, individuals in the high‐risk group had a subdistribution hazard ratio (sHR) of 16.73 (95% CI: 6.49–43.11, *p* < 0.001), indicating a markedly increased probability of developing ADRD over 5 years relative to the lower‐risk group, even when accounting for the competing risk of death (Figure 5).

### 3.6 Sensitivity and robustness analyses

We conducted multiple sensitivity analyses to evaluate the robustness of our integrated model. First, stratification by age at onset showed excellent discrimination for late‐onset ADRD (AUC = 0.915, 95% CI: 0.894–0.935) but substantially lower discrimination for early‐onset ADRD (AUC = 0.601, 95% CI: 0.552–0.654; *N* = 21), likely due to limited power and potential etiologic differences from late‐onset ADRD. Sex‐stratified analyses indicated consistent accuracy for women (AUC = 0.861) and men (AUC = 0.874). Excluding the *APOE* locus from the PRS modestly reduced performance of the multivariable model (AUC = 0.871 vs. 0.899). Stratification by *APOE‐ε4* status showed higher AUC in carriers (0.919) than non‐carriers (0.865); *APOE‐ε4* carrier status was used for stratification only. Removing lifestyle covariates lowered AUC modestly (0.847 vs. 0.899). Prediction remained largely consistent across time horizons (AUC = 0.893 for 5 years; 0.899 for 9 years) and across missing‐data strategies (complete‐case vs. imputation: AUC = 0.899 vs. 0.879) (Figure S9). Across 100 repeated stratified 70/30 splits, stepwise model performance remained stable, and a logistic‐regression benchmark reproduced the same qualitative improvement pattern with richer predictor sets (Tables S5 and S6).

In unadjusted (univariate) analyses, cause‐specific Cox models treating death as censoring and Fine–Gray competing‐risk models produced highly consistent effect estimates. PRS showed the strongest association with incident ADRD (Cox HR per SD = 3.13, 95% CI: 2.83–3.47; Fine–Gray sHR per SD = 3.11, 95% CI: 2.81–3.44). Most BA measures showed similar magnitudes across models (e.g., PhenoAge: HR = 2.82 vs. sHR = 2.48; MetaboAge: HR = 2.70 vs. sHR = 2.55), and TL was inversely associated with ADRD risk (HR = 0.72, 95% CI: 0.61–0.85; sHR = 0.74, 95% CI: 0.63–0.87) (Table S7).

Benchmarking against simpler predictor sets showed that the clinically feasible reference model including age, sex, lifestyle covariates, and PRS achieved AUC = 0.861 and AUPRC = 0.176 in the held‐out test set. The fully integrated Model 5 improved performance to AUC = 0.899 and AUPRC = 0.242, corresponding to ΔAUC = +0.038 and ΔAUPRC = +0.066 relative to the reference model. Supplementary reclassification metrics showed consistent incremental improvement (Table S8).

To assess model robustness for omics‐based BA clocks, we compared the stacked ensemble approach for ProtAge against advanced single‐model algorithms including XGBoost, CatBoost, and LightGBM. Across both sexes, stacking consistently outperformed all base learners, achieving the highest correlation with CA and the lowest RMSE and MAE (Table S9).

As a robustness check, we repeated the competing‐risk analysis after excluding individuals with very early follow‐up events (≤100 days). The cumulative incidence curves and effect estimates were essentially unchanged (sHR = 16.74, 95% CI: 6.50–43.14, *p* < 0.001), indicating that the observed risk separation was not driven by early events (Figure S10).

### 3.7 External validation in TwinGene

In external validation using TwinGene (*N* = 3772; 331 incident ADRD cases), the harmonized XGBoost framework showed moderate discrimination for incident ADRD. Predictors were harmonized to mirror the UKB Model 5 structure where possible (Table S10). Across 50 repeated stratified 70/30 splits, the final harmonized model achieved mean ROC‐AUC = 0.757 (empirical 2.5th–97.5th percentiles: 0.706 –0.794) and mean AUPRC = 0.223 (0.171–0.265) (Table S11). Performance was lower than in the UKB held‐out test set but remained above chance. The largest precision–recall gain was observed after adding genetic and biological‐aging predictors, with XGBoost AUPRC increasing from 0.196 for age + sex to 0.223 after inclusion of ADRD PRS, clinical biological aging measures, ProtAge, and MetaboAge. Logistic‐regression replication showed a similar pattern, with AUPRC increasing from 0.174 for age + sex to 0.222 after adding the full predictor set (Table S11). In a separate five‐fold out‐of‐fold full‐model analysis, the full TwinGene model achieved ROC‐AUC = 0.757 (95% CI: 0.706–0.794) and AUPRC = 0.223 (95% CI: 0.171–0.265) (Figure S11). Risk stratification was also preserved: participants in the top 25% of out‐of‐fold predicted risk had a higher ADRD event proportion than those in the lower 75% (19.9% vs. 5.1%) and showed greater cumulative incidence of ADRD in Fine–Gray competing‐risk analysis accounting for death as a competing event (sHR = 4.02, 95% CI: 3.25–5.02, *p *< 0.001; Table S12 and Figure S12).

## 4 DISCUSSION

In this large population‐based study, we developed and evaluated an integrative framework combining genetic risk with clinical, proteomic, and metabolomic biological aging measures to predict incident ADRD. The findings suggest that inherited susceptibility and systemic biological aging capture complementary dimensions of ADRD risk, with the largest incremental gains observed after adding genetic and proteomic‐aging signals. However, the magnitude and practical meaning of this improvement should be interpreted in relation to simpler baselines and the low ADRD event rate. Our findings mirror earlier work demonstrating that BA measures capture physiological changes linked to higher dementia risk^5^, ^6^ and overlap with processes frequently altered in neurodegenerative conditions,^1^, ^9^, ^10^ helping explain why these markers improve prediction. In the held‐out test set, the fully integrated model improved performance beyond a clinically feasible age/sex/lifestyle/PRS reference model (ΔAUC = +0.038; ΔAUPRC = +0.066; IDI = +0.039; continuous NRI = +0.202; Table S8), while also showing that simpler models captured a substantial proportion of the predictive signal. Accordingly, we interpret the model primarily as supporting risk stratification and prevention‐oriented enrichment rather than clinical classification at a universal default threshold. This interpretation is supported by AUPRC, calibration, decision‐curve analysis, threshold‐based operating characteristics, external validation, and repeated‐split sensitivity analyses. External validation/replication in TwinGene showed lower but above‐chance discrimination, consistent with partial transportability while highlighting expected performance attenuation across cohorts and omics/harmonization settings.

The ML prediction models and Fine–Gray analyses address complementary questions. Discrimination evaluates how well the combined predictor set ranks future ADRD cases versus non‐cases and may benefit from correlated predictors and nonlinear effects. In contrast, Fine–Gray coefficients estimate adjusted subdistribution associations for individual predictors conditional on the remaining variables and can be attenuated by collinearity and limited event counts. Thus, a predictor may improve model discrimination without remaining independently significant in the fully adjusted competing‐risk model. These analyses support prediction and association assessment, but not causal inference.

Importantly, discrimination metrics such as ROC‐AUC do not directly translate to clinical utility, particularly in a low‐incidence setting. Operating thresholds must be selected according to the intended use (e.g., trial enrichment vs. clinical screening), balancing sensitivity, specificity, and the consequences of false positives and false negatives. Although TwinGene provided independent external validation, additional validation in more diverse populations, ancestry groups, and omics‐platform settings—and prospective evaluation of calibration and decision‐analytic utility—is still required before clinical translation. Accordingly, the benchmarking and decision‐curve analyses should be interpreted as evidence of improved risk enrichment, not as sufficient evidence for immediate clinical deployment.

PRS remained a dominant contributor to ADRD prediction, consistent with extensive genetic evidence linking polygenic burden to dementia risk.^11^, ^26^, ^28^ CA and sex also remained influential, whereas clinical biological aging measures such as PhenoAge, FI, and TL contributed more modest incremental information. These findings support the view that systemic physiological decline and inherited susceptibility provide complementary, but partly overlapping, information about ADRD vulnerability.^1^, ^5^, ^6^, ^9^, ^16^ The attenuation of some aging measures in Fine–Gray models further highlights the importance of accounting for competing mortality and collinearity among aging‐related predictors.^43^

ProtAge contributed the largest incremental gain among the omics‐based aging clocks, but its biological interpretation requires caution.^14^, ^16^ ProtAge was trained to predict chronological age rather than ADRD or neurodegeneration and should therefore be interpreted as an age‐related proteomic summary rather than a disease‐specific or mechanistic biomarker. Its association with incident ADRD may reflect general biological aging, inflammation, vascular or metabolic dysregulation, frailty‐related physiology, and/or latent preclinical disease. Several proteins within the broader proteomic space, including GFAP, NEFL, and GDF15, are established dementia‐associated biomarkers reflecting astroglial activation, neuroaxonal injury, and cellular stress responses.^1^, ^9^, ^14^, ^57^ Therefore, the predictive contribution of ProtAge may partly capture early disease‐related biology rather than “aging acceleration” alone. Although risk separation remained robust after excluding very early events (≤100 days), this does not exclude longer prodromal processes. Accordingly, we interpret ProtAge as a risk‐enrichment feature rather than evidence of a specific causal aging mechanism. MetaboAge showed strong univariate associations with ADRD, but its contribution attenuated after adjustment for PRS and other biological aging measures, suggesting that metabolomic aging may capture complementary but less independent lipid/metabolic physiology once genetic risk and other aging dimensions are modeled jointly.^15^, ^37^, ^38^, ^58^

Strengths of this study include the integration of diverse BA clocks with genomic risk and lifestyle factors within a unified framework, providing a comprehensive systems‐level view of ADRD susceptibility. We evaluated both discrimination and subdistribution hazard ratios, ensuring relevance for risk prediction in older populations. Extensive sensitivity analyses, including sex, age‐at‐onset, *APOE* exclusion, excluding lifestyle covariates, prediction horizons, as well as missing‐data strategies demonstrated consistent performance.

This study has important limitations. The analytic cohort required complete biomarker and omics measures and therefore represents a subset of the eligible UKB sample. However, included and excluded participants were similar across baseline characteristics (all standardized mean differences < 0.10; Table S1), and model discrimination was robust to missing‐data handling (Figure S9). The cohort was predominantly of White‐European ancestry, limiting generalizability, particularly for PRS and omics‐based aging measures.^26^, ^28^, ^39^ Single time‐point omics measurements limit assessment of BA trajectories. UKB's healthier volunteer bias may attenuate associations, particularly for metabolomic and frailty measures. Early‐onset ADRD prediction was limited by the small number of cases (*N* = 21). Another important limitation is outcome heterogeneity: ADRD was analyzed as a broad composite endpoint, including Alzheimer's disease, vascular or mixed dementia, and other or unspecified dementia. This improves event capture but may obscure subtype‐specific etiologies. In our recent UKB PRS study, subtype‐specific analyses showed stronger PRS discrimination for Alzheimer's disease and somewhat lower discrimination for vascular and mixed/unspecified dementia, supporting subtype heterogeneity.^21^ In the present multi‐omics complete‐case sample, subtype‐specific modeling was not pursued because case numbers within individual subtype strata were limited and registry‐coded diagnoses may reflect diagnostic overlap, mixed pathology, and ascertainment variability. Therefore, the observed performance should be interpreted as prediction of broad ADRD risk rather than Alzheimer's disease–specific prediction. Omics aging clocks capture systemic rather than brain‐specific biology, and ProtAge may partly reflect dementia‐related plasma biomarkers or latent preclinical disease rather than aging acceleration alone. We also did not separately model medication use, hypertension, diabetes, or cardiovascular disease as sensitivity analyses; these factors are clinically relevant but may overlap with or mediate biological‐aging signals, and detailed medication histories may introduce confounding by indication. Future studies should evaluate whether harmonized cardiometabolic diagnoses, treatment histories, repeated omics measurements, and biomarker‐defined dementia subtypes improve calibration, clinical utility, and transportability beyond multi‐omics aging and genetic risk.

Our findings support further evaluation of combining PRS with multi‐omics–derived biological aging measures for ADRD risk stratification. The incremental improvement over simpler predictor sets suggests potential value for prevention‐oriented enrichment, while cost‐effectiveness, clinical thresholds, prospective calibration, and decision‐analytic utility require additional study. As proteomic and clinical biomarker assays continue to scale, integration of biological aging clocks may become increasingly feasible in large observational cohorts and health‐system research settings. Future work should prioritize validation across diverse populations and omics platforms, repeated omics measurements to model aging trajectories, and integration with cognitive measures, digital phenotypes, and brain imaging. Ultimately, integrating systems‐level aging markers with genetic risk may help advance risk‐stratified prevention research for ADRD.

## AUTHOR CONTRIBUTIONS

Shayan Mostafaei and Sara Hägg contributed to the conceptualization and methodology of the study. Shayan Mostafaei, Sara Hägg, and Trung Nghia Vu were responsible for ML development, formal analysis, and data curation. Shayan Mostafaei, Hampus Hagelin, Jonathan K.L. Mak, and Ida K. Karlsson participated in the data preparation. Resources were provided by Sara Hägg. Shayan Mostafaei led the visualization and project administration. The original draft was written by Shayan Mostafaei, Karolina Gustavsson, and Sara Hägg, and all authors (Shayan Mostafaei, Karolina Gustavsson, Hampus Hagelin, Jonathan K. L. Mak, Trung Nghia Vu, Ida K. Karlsson, and Sara Hägg) contributed to review and editing. Supervision and funding acquisition were provided by Sara Hägg and Shayan Mostafaei. All authors have read and approved the final manuscript.

## CONFLICT OF INTEREST STATEMENT

The authors declare no conflicts of interest. Author disclosures are available in the Supporting Information.

## ETHICS APPROVAL

The UK Biobank study was approved by the North West Multi‐Centre Research Ethics Committee. The specific research aim was approved by the Swedish Ethical Review Authority (Dnr. 2022‐06634‐01). The TwinGene study was approved by the Regional Ethics Review Board, Stockholm, Sweden (Dnr. 2025‐02440‐02).

## CONSENT STATEMENT

All UK Biobank and TwinGene participants provided written informed consent. Analyses were conducted using fully de‐identified data.

## Supporting information



Supporting Information

ICMJE Disclosure Forms

## Data Availability

Data from the UK Biobank are available to bona fide researchers upon application via the UK Biobank Access Management System (https://www.ukbiobank.ac.uk/enable‐your‐research/apply‐for‐access). The TwinGene data are part of the Swedish Twin Registry and are not publicly available due to ethical and legal restrictions. Access to TwinGene data requires approval from the Swedish Ethical Review Authority and the Steering Committee of the Swedish Twin Registry. Researchers interested in accessing TwinGene data may contact the registry (str-research@meb.ki.se) for further information on the application process. Code used for data processing and analysis is available at:
DDML PRS construction: https://github.com/shayanmostafaei/DDML_PRS_ADRD
Metabolomic aging clock (MetaboAge): https://github.com/shayanmostafaei/Metabolomic‐Aging‐Clock‐MetaboAge‐
Proteomic aging clock (ProtAge): https://github.com/shayanmostafaei/Proteomic‐Aging‐Clock‐ProtAge‐
Integrated modeling pipeline (main analyses): https://github.com/shayanmostafaei/Omics_ADRD_Integrated_Modeling
