# Supplementary material for: Integrative prediction of Alzheimer's disease and related dementias using multi‐omics aging clocks and genetic data
Source: Alzheimers Dement. 2026 Sep 28;22(10):e71869. doi: 10.1002/alz.71869 (PMC13619731; doi:10.1002/alz.71869)
Supplement: Supplementary file 2 [file ALZ-22-e71869-s001.docx]

**Supplementary Appendix A**

**Supplementary Figures, Supplementary Tables, and Supplementary Methods**

**Manuscript**: *Integrative Prediction of Alzheimer’s Disease and Related Dementias Using Multi-Omics Aging Clocks and Genetic Data*

Shayan Mostafaei^1*^, Karolina Gustavsson^1^, Hampus Hagelin^1^, Jonathan K. L. Mak^1,2^, Trung Nghia Vu^1^, Ida K. Karlsson^1^, Sara Hägg^1*^

- 1. Department of Medical Epidemiology and Biostatistics, Karolinska Institutet, Stockholm, Sweden.
- 2. Department of Pharmacology and Pharmacy, Li Ka Shing Faculty of Medicine, The University of Hong Kong, Hong Kong, China

*Corresponding Authors:

1- Shayan Mostafaei, PhD, Department of Medical Epidemiology and Biostatistics, Karolinska Institutet, SE-17177 Solna, Sweden. Email: [shayan.mostafaei@ki.se](mailto:shayan.mostafaei@ki.se)

2- Sara Hägg, PhD, Department of Medical Epidemiology and Biostatistics, Karolinska Institutet, SE-17177 Solna, Sweden. Email: [sara.hagg@ki.se](mailto:sara.hagg@ki.se)

This Supplementary Appendix provides additional tables and figures supporting the main manuscript, as well as supplementary methods describing sensitivity analyses, robustness checks, and external validation.

**Contents**

**Supplementary Tables**

Table A.1. Characteristics of included versus excluded participants ………………………...………… p. 4

Table A.2. Associations of lifestyle/demographic factors with biological aging measures and PRS..… p. 5

Table A.3. Threshold-based operating characteristics of Model 5 in the UKB held-out test set ….…… p. 6

Table A.4. Alternative predictor-entry orders…………………………………………………………… p. 6

Table A.5. Stability of stepwise XGBoost models under 100 repeated stratified 70/30 splits…….……. p. 7

Table A.6. Benchmarking under 100 repeated stratified 70/30 splits using logistic regression…..….… p. 7

Table A.7. Univariate Cox and Fine–Gray associations with incident ADRD……………………….… p. 8

Table A.8. Benchmarking Model 5 against simpler clinically feasible predictor sets ….………...….… p. 8

Table A.9. Cross-validated age-prediction performance of ProtAge and MetaboAge……………..…… p. 9

Table A.10. TwinGene harmonized predictor availability and mapping to UKB Model 5……..……... p. 10

Table A.11. TwinGene external validation/replication performance…………………………….……. p. 11

Table A.12. TwinGene competing-risk risk stratification by predicted-risk group…………….……… p. 12

**Supplementary Figures**

Figure A.1. Distribution of PRS among participants with and without ADRD………………….….… p. 13

Figure A.2. Performance of ProtAge in UKB ………………………………………..……………..… p. 14

Figure A.3. Performance of MetaboAge in UKB ……….……………..……………………………… p. 15

Figure A.4. Associations between lifestyle factors and aging measures……………….……………… p. 16

Figure A.5. ROC and precision–recall curves for stepwise UKB ADRD prediction.….…..…..……… p. 17

Figure A.6. Calibration of Model 5 in the UKB held-out test set….………………………..….……… p. 18

Figure A.7. Decision curve analysis for Model 5 in the UKB held-out test set……………...…….….. p. 19

Figure A.8. SHAP summary plot for Model 5…………………………………………….…………… p. 20

Figure A.9. Sensitivity analyses of Model 5 ………………………………….………..……….…… p. 21

Figure A.10. Early-event exclusion sensitivity analysis …..………………………………………… p. 22

Figure A.11. TwinGene ROC and precision–recall curves ………………..……………..……….… p. 22

Figure A.12. TwinGene cumulative incidence by predicted-risk group…………………..……….… p. 23

**Supplementary Methods**

Methods A.M1. Sensitivity Analyses and Robustness Checks………………..……….….…..…… p. 24–30
Methods A.M2. External validation and replication in TwinGene………………….………..….… p. 30–33

***Table A.1*.** Characteristics of included versus excluded participants from the UK Biobank sample

| **Characteristics** | **Excluded Participants** | **Included Participants** | **SMD** |
| --- | --- | --- | --- |
| Age, years (mean ±SD) | 56.52 ± 8.10 | 56.98 ± 7.88 | 0.057 |
| *APOE-ε4* carriers (N, %) | 72768 (15.5%) | 2423 (15%) | 0.014 |
| Sex, men (N, %) | 215506 (45.8%) | 7422 (45.8%) | <0.001 |
| Deaths during follow-up (N, %) | 41222 (8.8%) | 1510 (9.3%) | 0.017 |
| ADRD PRS (mean ± SD) | -0.46 ± 1.39 | -0.59 ± 1.30 | 0.097 |
| PhenoAge, years (mean ±SD) | 50.30 ± 9.35 | 50.88 ± 9.26 | 0.062 |
| Klemera-Doubal Method, years (mean ±SD) | 55.23 ± 8.84 | 55.76 ± 8.74 | 0.060 |
| Homeostatic dysregulation (mean ±SD) | 2.26 ± 1.03 | 2.18 ± 0.87 | 0.078 |
| Frailty Index (mean ±SD) | 0.11 ± 0.08 | 0.11 ± 0.07 | 0.024 |
| Telomere length (mean ±SD) | 0.83 ± 0.13 | 0.83 ± 0.14 | 0.011 |
| ProtAge, years (mean ±SD) | 56.76 ± 8.06 | 56.97 ± 7.75 | 0.027 |
| MetaboAge, years (mean ±SD) | 56.79 ± 7.36 | 56.94 ± 5.85 | 0.023 |
| Body mass index, kg/m^2^ (mean ±SD) | 27.33 ± 4.72 | 27.32 ± 4.52 | 0.002 |
| Alcohol intake frequency, ordinal categories (mean ±SD) | 2.91 ± 1.66 | 2.88 ± 1.53 | 0.019 |
| Smokers, current or former (N, %) | 227115 (45.8%) | 7253 (44.7%) | 0.021 |
| Education level, high or intermediate (N, %) | 344568 (84%) | 13253 (81.7%) | 0.061 |

Included participants were those in the analytic cohort with complete biomarker/omics data (n = 16,215); excluded participants comprised the remaining eligible UK Biobank participants not included in the analytic cohort. Values are mean (SD) or n (%). Group differences are summarized using standardized mean differences (SMD), where SMD < 0.10 indicates negligible imbalance. *APOE-ε4* carriers: Number and percentage of participants carrying at least one *APOE-ε4* allele. High or intermediate education is defined as ≥10 years of formal education. Smoking status includes both current and former smokers. Alcohol intake frequency is UK Biobank Field 1558 (ordinal categories 1–7: daily or almost daily to never); values are shown as mean ± SD. Biological aging measures include Phenotypic Age (PhenoAge), Klemera-Doubal Method (KDM), Homeostatic Dysregulation (HD), Frailty Index (FI), and adjusted Telomere Length (TL; UK Biobank leukocyte telomere length adjusted for technical/assay parameters). FI was calculated as a 49-item Rockwood frailty index (range from 0–1), representing the proportion of deficits present. Polygenic Risk Scores (PRS) for ADRD are calculated using Bayesian variational autoencoder method including the *APOE* region. PRS values are shown on the original scale used in the model (higher values indicate higher genetic risk).

***Table A.2*.** Associations of lifestyle/demographic factors with biological aging measures and PRS (linear regression; FDR controlled using Benjamini–Hochberg) in UK Biobank

| **Predictor** | **BMI**  **(Per kg/m²)** | **Smoking:**  **Current or former (vs Non-smoker)** | **Alcohol intake frequency (Per category)** | **Education: Low (vs High)** | **Sex: Men (vs Women)** |
| --- | --- | --- | --- | --- | --- |
| **KDM** | 0.277 (9.0×10⁻^46^) | 0.536 (9.5×10⁻^05^) | −0.073 (0.227) | 6.983 (4.4×10⁻^147^) | −1.060 (4.2×10⁻^09^) |
| **PhenoAge** | 0.252 (5.4×10⁻^34^) | 1.037 (6.5×10⁻^13^) | 0.119  (0.068) | 6.384 (2.8×10⁻^110^) | 1.958 (1.0×10⁻^24^) |
| **ProtAge** | 0.090 (3.7×10⁻^07^) | 0.297  (0.018) | −0.092 (0.092) | 5.638 (3.5×10⁻^119^) | 0.308  (0.068) |
| **MetaboAge** | 0.157 (9.9×10⁻^32^) | 0.330 (4.9×10⁻^04^) | −0.130 (0.002) | 3.225 (1.6×10⁻^68^) | 0.350  (0.006) |
| **HD** | 0.052 (5.9×10⁻^170^) | 0.051 (9.8×10⁻^05^) | 0.024 (3.8×10⁻^05^) | 0.257 (1.4×10⁻^23^) | −0.465 (6.8×10⁻^159^) |
| **FI** | \| 0.003 (2.2×10⁻^103^) | 0.012 (3.2×10⁻^29^) | −0.004 (1.3×10⁻^19^) | 0.028 (5.5×10⁻^36^) | −0.009 (4.6×10⁻^10^) |
| **TL** | −0.001  (0.068) | −0.002 (0.498) | −0.002  (0.092) | −0.038 (9.6×10⁻^17^) | −0.028 (1.3×10⁻^11^) |
| **PRS** | −0.005  (0.111) | −0.028  (0.208) | −0.004 (0.696) | 0.098  (0.028) | 0.024  (0.417) |

Cells show *β* (FDR q-value). *β* coefficients are from linear regression models evaluating associations between lifestyle/demographic factors (body mass index, smoking, alcohol intake frequency, education, sex) and each biological aging measure or PRS. Alcohol was modeled as an ordinal variable (“per category” increase) to provide a single effect estimate per exposure. FDR: Benjamini–Hochberg false discovery rate (q-value). Education reference category: High; sex reference category: Women; smoking reference category: Non-smoker. Alcohol intake frequency corresponds to UK Biobank Field 1558 (ordinal categories 1–7: daily or almost daily to never). KDM: Klemera–Doubal Method; HD: homeostatic dysregulation; ProtAge: proteomic aging clock; MetaboAge: metabolomic aging clock; FI: frailty index; TL: leukocyte telomere length; PRS: polygenic risk score.

***Table A.3*:** Threshold-based operating characteristics of the final integrated ADRD prediction model in the held-out test set (UK Biobank)

| Operating point | Probability threshold | TP | FP | FN | TN | Sensitivity | Specificity | PPV | NPV | F1 | Accuracy |
| --- | --- | --- | --- | --- | --- | --- | --- | --- | --- | --- | --- |
| Top 5% predicted risk (test) | 0.280 | 39 | 205 | 83 | 4,537 | 0.320 | 0.957 | 0.160 | 0.982 | 0.213 | 0.941 |
| Top 10% predicted risk (test) | 0.108 | 60 | 427 | 62 | 4,315 | 0.492 | 0.910 | 0.123 | 0.986 | 0.197 | 0.899 |
| Illustrative default threshold 0.5 (test) | 0.500 | 32 | 105 | 90 | 4,637 | 0.262 | 0.978 | 0.234 | 0.981 | 0.247 | 0.960 |

Values are computed for the final XGBoost model (Model 5) evaluated in the held-out test set from the stratified 70/30 train–test split. The held-out test set included 4,864 participants, with 122 incident ADRD cases and 4,742 non-cases. The top 5% and top 10% predicted-risk thresholds correspond to the 95th and 90th percentiles of predicted risk in the test set, respectively. The 0.5 threshold is included only as an illustrative default probability threshold and was not optimized for this low-incidence outcome. Metrics are based on the 2×2 confusion matrix: TP = true positives, FP = false positives, FN = false negatives, TN = true negatives; sensitivity = TP/(TP+FN), specificity = TN/(TN+FP), PPV = TP/(TP+FP), NPV = TN/(TN+FN), F1 = 2·PPV·sensitivity/(PPV+sensitivity), and accuracy = (TP+TN)/N.

***Table A.4*.** Sensitivity analysis: final discrimination of the fully integrated model (Model 5) under alternative predictor-entry orders (UK Biobank)

| **Ordering** | **Predictor blocks added (sequence)** | **AUC (95% CI)** | $\boldsymbol{\Delta}$**AUC vs order A** | **DeLong *P* vs order A** |
| --- | --- | --- | --- | --- |
| **A (primary)** | Age+Sex → Lifestyle → PRS → BA core → ProtAge → MetaboAge | 0.899 (0.863–0.926) | Ref. | Ref. |
| **B** | Age+Sex → Lifestyle → PRS → ProtAge → BA core → MetaboAge | 0.901 (0.867–0.935) | +0.002 | 0.708 |
| **C** | Age+Sex → Lifestyle → BA core → PRS → ProtAge → MetaboAge | 0.904 (0.872–0.937) | +0.005 | 0.290 |
| **D** | Age+Sex → Lifestyle → PRS → BA core → (ProtAge+MetaboAge together) | 0.903 (0.871–0.936) | +0.004 | 0.299 |

Alternative stepwise entry orders were evaluated on the same stratified 70/30 train–test split using a complete-case subset across all predictors. Final AUCs were computed in the held-out test set; 95% CIs were obtained via DeLong. BA = PhenoAge + frailty index + telomere length; Lifestyle = smoking + alcohol intake frequency + body mass index + education.

***Table A.5.*** Stability of stepwise XGBoost models under 100 repeated stratified 70/30 splits in UK Biobank

| **Model** | **ROC AUC (mean [2.5–97.5%])** | **AUPRC (mean [2.5–97.5%])** | **reps_used** |
| --- | --- | --- | --- |
| Base: Age + Sex | 0.787 [0.751–0.807] | 0.066 [0.027–0.100] | 100 |
| M1: + Lifestyle | 0.817 [0.774–0.834] | 0.089 [0.067–0.102] | 100 |
| M2: + PRS | 0.857 [0.842–0.872] | 0.177 [0.156–0.252] | 100 |
| M3: + BA | 0.881 [0.825–0.899] | 0.201 [0.155–0.254] | 100 |
| M4: + ProtAge | 0.895 [0.882–0.910] | 0.222 [0.168–0.287] | 100 |
| M5: + MetaboAge | 0.903 [0.881–0.920] | 0.239 [0.190–0.288] | 100 |

Models correspond to the stepwise XGBoost sequence in Table 2. We performed 100 repeated, reproducible stratified 70/30 train–test splits stratified by incident ADRD status, sex, and baseline age bin (5-year bins). Each repeat used a deterministic replicate-specific seed derived from a fixed master seed. All preprocessing and model fitting (including omics-clock derivation where applicable) were performed in the training split only, and all metrics were computed once on the held-out test split. Values are reported as mean [empirical 2.5th–97.5th percentiles] across repeats. reps_used indicates the number of repeats successfully completed.

***Table A.6.*** Benchmarking under 100 repeated stratified 70/30 splits using logistic regression in UK Biobank

| **Benchmark_model** | **ROC AUC (mean [2.5–97.5%])** | **AUPRC (mean [2.5–97.5%])** | **reps_used** |
| --- | --- | --- | --- |
| Base: Age + Sex | 0.791 [0.783–0.800] | 0.071 [0.033–0.101] | 100 |
| Full: Model 5 | 0.868 [0.853–0.883] | 0.228 [0.181–0.291] | 100 |

Benchmark models were fit using logistic regression on the training split and evaluated on the held-out test split under the same 100 repeated stratified 70/30 splits described in Table A.5 (stratified by ADRD status, sex, and 5-year age bins; replicate-specific deterministic seeds from a fixed master seed). The “Base” model includes age and sex; the “Full” model uses the Model 5 predictor set. Values are reported as mean [empirical 2.5th–97.5th percentiles] across repeats. reps_used indicates the number of repeats successfully completed.

***Table A.7*.** Univariate associations with incident ADRD: Cause-specific Cox (death censored) vs Fine–Gray competing-risk models in UK Biobank (Time scale: follow-up time since baseline (days); effects per 1 SD increase in each predictor**)**

| **Predictor** | **Cox HR (95% CI)** | **Fine–Gray sHR (95% CI)** |
| --- | --- | --- |
| **PRS** | 3.13 (2.83–3.47) | 3.11 (2.81–3.44) |
| **CA** | 3.51 (2.24–5.17) | 3.15 (1.92–4.76) |
| **ProtAge** | 3.70 (2.34–5.50) | 3.30 (1.99–5.03) |
| **MetaboAge** | 2.70 (2.25–3.23) | 2.55 (2.12–3.07) |
| **PhenoAge** | 2.82 (2.44–3.26) | 2.48 (2.22–2.76) |
| **KDM** | 3.22 (2.73–3.81) | 3.15 (2.22–4.26) |
| **HD** | 1.26 (1.14–1.38) | 1.19 (1.09–1.31) |
| **FI** | 1.42 (1.26–1.61) | 1.40 (1.25–1.57) |
| **TL** | 0.72 (0.61–0.85) | 0.74 (0.63–0.87) |

Values are from unadjusted (univariate) models of incident Alzheimer’s disease and related dementias (ADRD). Cox models are cause-specific hazards treating death as censoring (HR: hazard ratio). Fine–Gray models account for death as a competing event (subdistribution hazard ratio; sHR). The time scale is “time since baseline assessment”, measured in days. Effect sizes are reported per 1 SD increase in each predictor. CA = chronological age; PRS = polygenic risk score; ProtAge = proteomic aging clock; MetaboAge = metabolomic aging clock; PhenoAge = phenotypic age; KDM: Klemera–Doubal Method; HD: homeostatic dysregulation; FI = frailty index; TL = leukocyte telomere length.

***Table A.8.*** Benchmarking the final integrated ADRD prediction model against simpler and clinically feasible predictor sets in the UK Biobank held-out test set.

| **Model** | **ROC AUC (95% CI)** | **AUPRC (95% CI)** | **Delta AUC vs reference** | **Delta AUPRC vs reference** | **IDI vs reference** | **Continuous NRI vs reference** |
| --- | --- | --- | --- | --- | --- | --- |
| Age + sex | 0.793 (0.762-0.824) | 0.065 (0.053-0.094) | -0.068 | -0.111 | -0.088 | -0.382 |
| Age + sex + PRS | 0.841 (0.805–0.875) | 0.160 (0.118–0.227) | -0.020 | -0.016 | -0.032 | -0.272 |
| Age + sex + PRS + ProtAge | 0.836 (0.801–0.871) | 0.172 (0.124–0.255) | -0.025 | -0.004 | -0.032 | -0.274 |
| Age + sex + PRS + PhenoAge + FI + TL | 0.842 (0.808–0.877) | 0.190 (0.135–0.267) | -0.019 | +0.014 | -0.019 | -0.221 |
| Age + sex + PRS + ProtAge + MetaboAge | 0.828 (0.787–0.868) | 0.177 (0.129–0.247) | -0.033 | +0.001 | -0.035 | -0.256 |
| Age + sex + lifestyle + PRS | 0.861 (0.832-0.891) | 0.176 (0.125-0.266) | Ref. | Ref. | Ref. | Ref. |
| Full Model 5 | 0.899 (0.863–0.926) | 0.242 (0.165-0.344) | +0.038 | +0.066 | +0.039 | +0.202 |

Values are based on the held-out test split from the same stratified 70/30 train–test partition used for the predictor-set benchmarking analysis. Models were fitted using the XGBoost pipeline and compared with a clinically feasible reference model consisting of age, sex, lifestyle covariates, and PRS including the *APOE* locus. *APOE* genotype was not entered separately; *APOE* information was incorporated through the *PRS* including the *APOE* locus. ROC AUC and AUPRC are reported with 95% confidence intervals. ΔAUC and ΔAUPRC are calculated relative to the reference model. IDI and continuous NRI are reported as supplementary reclassification metrics relative to the reference model; positive values indicate improved average discrimination or category-free reclassification compared with the reference model. PRS = polygenic risk score; AUPRC = area under the precision–recall curve; IDI = integrated discrimination improvement; NRI = net reclassification improvement.

***Table A.9*.** Cross-validated age-prediction performance of ProtAge and MetaboAge, stratified by sex in UK Biobank

| **ProtAge** | **Women** | | | **Men** | | |
| --- | --- | --- | --- | --- | --- | --- |
| **Model** | Correlation | RMSE | MAE | Correlation | RMSE | MAE |
| XGBoost | 0.8941 | 3.7088 | 2.9011 | 0.8894 | 3.8008 | 2.9605 |
| CatBoost | 0.9000 | 3.5982 | 2.8133 | 0.8953 | 3.6963 | 2.8924 |
| LightGBM | 0.9035 | 3.5547 | 2.7725 | 0.8981 | 3.6413 | 2.8423 |
| Stacking | 0.9715 | 1.9299 | 1.3084 | 0.9748 | 1.8734 | 1.2247 |
| **MetaboAge** | **Women** | | | **Men** | | |
| **Model** | Correlation | RMSE | MAE | Correlation | RMSE | MAE |
| XGBoost | 0.6359 | 6.0043 | 4.9451 | 0.6008 | 6.3468 | 5.2554 |
| CatBoost | 0.6972 | 5.5890 | 4.6021 | 0.6816 | 5.8521 | 4.8385 |
| LightGBM | 0.6376 | 5.9924 | 4.9345 | 0.6097 | 6.3038 | 5.2191 |
| Stacking | 0.7443 | 5.1739 | 4.1893 | 0.7340 | 5.4195 | 4.3992 |

Values are cross-validated performance metrics computed within the training split only. Correlation refers to the Pearson correlation between predicted age and chronological age. RMSE and MAE are reported in years (lower values indicate better performance). Models compared include XGBoost, CatBoost, LightGBM, and a stacked ensemble (“Stacking”) combining base learners. Sex-stratified results were obtained by fitting and evaluating models separately in women and men to assess potential differences in predictive accuracy across sexes.

***Table A.10***. Harmonized TwinGene predictors mapped to the UK Biobank Model 5 framework

| **Domain** | **UKB Model 5 variable** | **TwinGene harmonized variable** | **Included in TwinGene full model** | **Notes** |
| --- | --- | --- | --- | --- |
| Demographics | Chronological age | Age | Yes | Baseline age |
| Demographics | Sex | Sex | Yes | Male indicator |
| Lifestyle | BMI | BMI | Yes | Continuous |
| Lifestyle | Smoking | Smoking | Yes | Harmonized smoking status |
| Lifestyle | Alcohol intake | Alcohol intake | Yes | Harmonized alcohol frequency |
| Education | Education | Education in years | Yes | Years of education |
| Genetics | ADRD PRS including *APOE* | PRS_ADRD | Yes | Same SNP panel/weights as UKB; 73 SNPs available |
| Ancestry | PC1–PC10 | PC1–PC10 | Yes | Genetic PCs |
| Genetics / sensitivity | *APOE ε4* carrier status | *APOEe4*_status | No | Not entered separately; *APOE* information incorporated through ADRD PRS |
| Genetics / sensitivity | PRS without *APOE* | PRS_ADRD_Without_APOE | Sensitivity only | *APOE*-excluded PRS available for sensitivity analyses |
| Clinical BA | PhenoAge | PhenoAge | Yes | Clinical biological-aging measure; standardized within TwinGene |
| Clinical BA | Frailty index | FI | Yes | Deficit-accumulation frailty index; standardized within TwinGene |
| Clinical BA | Telomere length | TL | Yes | Leukocyte telomere length; standardized within TwinGene |
| Clinical BA/Sensitivity | KDM | KDM | Sensitivity / correlation only | Not included in primary full model to reduce redundancy |
| Clinical BA/Sensitivity | HD | HD | Sensitivity / correlation only | Not included in primary full model to reduce redundancy |
| Proteomic aging | ProtAge | ProtAge | Yes | TwinGene-derived/protein-based ProtAge predictor |
| Metabolomic aging | MetaboAge | MetaboAge | Yes | TwinGene-aligned MetaboAge predictor; standardized within TwinGene |

TwinGene predictors were harmonized to mirror the UK Biobank Model 5 predictor framework where possible. Continuous variables were standardized within TwinGene before model fitting. The covariate-augmented TwinGene full model included chronological age, sex, BMI, smoking, alcohol intake, education, ADRD PRS including the *APOE* locus, genetic principal components PC1–PC10, PhenoAge, frailty index, telomere length, ProtAge, and MetaboAge. Categorical variables were dummy-coded using model-matrix encoding. *APOE ε4* carrier status was not entered separately; *APOE* information was incorporated through the ADRD PRS. KDM, HD, PRS without *APOE*, and *APOE ε4* carrier status were reserved for sensitivity or descriptive analyses. Because many variables were not measured identically across UK Biobank and TwinGene, this analysis is interpreted as a cohort-harmonized methodological replication rather than direct deployment of a frozen UKB-trained model. BA = biological aging; ADRD = Alzheimer’s disease and related dementias; PRS = polygenic risk score; FI = frailty index; TL = telomere length; KDM = Klemera–Doubal Method; HD = homeostatic dysregulation.

***Table A.11***. External validation and replication performance of harmonized ADRD prediction models in TwinGene

| **Model** | **Predictor set** | **XGBoost**  **ROC AUC mean [2.5–97.5%]** | **XGBoost**  **AUPRC mean [2.5–97.5%]** | **Logistic**  **ROC AUC mean [2.5–97.5%]** | **Logistic**  **AUPRC mean [2.5–97.5%]** |
| --- | --- | --- | --- | --- | --- |
| Base | Age + sex | 0.753 [0.723-0.785] | 0.196 [0.149-0.238] | 0.736 [0.701-0.782] | 0.174 [0.138-0.210] |
| Model 1 | Base + lifestyle | 0.748 [0.710-0.780] | 0.191 [0.147-0.235] | 0.704 [0.667-0.743] | 0.169 [0.131-0.203] |
| Model 2 | Model 1 + PRS | 0.753 [0.709-0.787] | 0.211 [0.159-0.252] | 0.730 [0.682-0.773] | 0.202 [0.153-0.247] |
| Model 3 | Model 2 + PhenoAge + FI + TL | 0.753 [0.709-0.789] | 0.215 [0.160-0.257] | 0.739 [0.703-0.773] | 0.211 [0.166-0.262] |
| Model 4 | Model 3 + ProtAge | 0.756 [0.704-0.791] | 0.220 [0.169-0.263] | 0.747 [0.710-0.785] | 0.221 [0.178-0.283] |
| Model 5 | Model 4 + MetaboAge | 0.757 [0.706-0.794] | 0.223 [0.171-0.265] | 0.747 [0.710-0.782] | 0.222 [0.170-0.279] |

Values summarize performance across 50 repeated stratified 70/30 train–test splits in TwinGene and are reported as mean [empirical 2.5th–97.5th percentiles] across repeats, not as formal confidence intervals. Continuous predictors were standardized within each TwinGene training split and applied to the corresponding held-out test split. The full harmonized model included age, sex, BMI, smoking, alcohol intake, education, ADRD PRS including the *APOE* locus, PC1–PC10, PhenoAge, frailty index, telomere length, ProtAge, and MetaboAge. XGBoost models used internal 5-fold cross-validation for early stopping within each training split; logistic-regression models used binomial GLM. Figure A.11 displays the ROC and precision–recall curves from the separate 5-fold out-of-fold full-model prediction analysis used for risk stratification, whereas this table summarizes repeated-split performance stability across the stepwise predictor sequence. Results are interpreted as cohort-harmonized methodological replication and predictor-set validation, not direct deployment of a frozen UKB-trained model. ADRD = Alzheimer’s disease and related dementias; PRS = polygenic risk score; FI = frailty index; TL = telomere length; AUPRC = area under the precision–recall curve.

***Table A.12***. TwinGene competing-risk risk stratification by out-of-fold predicted ADRD risk

| **Risk group** | **N** | **N of ADRD event** | **N of death-before-ADRD** | **N of censored** | **ADRD event proportion** | **Fine–Gray sHR** | **95% CI** | ***P*-value** |
| --- | --- | --- | --- | --- | --- | --- | --- | --- |
| Lower 75% predicted risk | 2,829 | 143 | 483 | 2,203 | 5.1% | Ref. | Ref. | Ref. |
| Top 25% predicted risk | 943 | 188 | 358 | 397 | 19.9% | 4.02 | 3.25–5.02 | <0.001 |

Risk groups were defined using out-of-fold predicted risks from the covariate-augmented full TwinGene model, including age, sex, BMI, smoking, alcohol intake, education, ADRD PRS including the *APOE* locus, PC1–PC10, PhenoAge, frailty index, telomere length, ProtAge, and MetaboAge. Predicted risks were generated using 5-fold cross-fitting so that each participant’s risk score was obtained from a model not trained on that participant. Fine–Gray competing-risk regression compared the top 25% versus lower 75% of predicted risk, treating death before ADRD as a competing event. sHR = subdistribution hazard ratio; CI = confidence interval; ADRD = Alzheimer’s disease and related dementias.

***Figure A.1.* Distribution of polygenic risk scores (PRS) among participants with and without ADRD**. Violin plot depicting PRS values for participants with ADRD during follow-up (N = 397) and ADRD-free participants throughout follow-up (N = 15,818) in the UK Biobank cohort, illustrating higher median scores and greater variability among participants with ADRD.

***Figure A.2.* Performance of the proteomic aging clock (ProtAge) in the clock-development dataset***.* Scatter plot showing the relationship between ProtAge and chronological age in UK Biobank participants (N = 50,482). The grey identity line (y = x) indicates perfect agreement between predicted and chronological age. The solid black line represents the linear regression fit, and the dashed black line shows a LOESS-smoothed trend to visualize potential non-linearity. Pearson’s correlation coefficient (*r* = 0.97) and root mean squared error (RMSE = 1.95) indicate high predictive accuracy. ProtAge: proteomic aging clock; LOESS: locally estimated scatterplot smoothing; RMSE: root mean squared error.

***Figure A.3.* Performance of the metabolomic aging clock (MetaboAge) in the clock-development dataset**. Scatter plot showing the relationship between MetaboAge and chronological age in UK Biobank participants (N = 228,513). The grey identity line (y = x) indicates perfect agreement between predicted and chronological age. The solid black line represents the linear regression fit, and the dashed black line shows a LOESS-smoothed trend to visualize potential non-linearity. Pearson’s correlation coefficient (*r* = 0.74) and root mean squared error (RMSE = 5.28) indicate moderate predictive accuracy. MetaboAge: metabolomic aging clock; LOESS: locally estimated scatterplot smoothing; RMSE: root mean squared error.

***Figure A.4.* Associations between lifestyle/demographic factors, aging measures, and polygenic risk score.** Heatmap of standardized regression coefficients (Std. *β*) from linear regression models assessing associations between lifestyle/demographic factors (BMI, smoking, alcohol intake frequency, education, and sex) and aging measures or PRS, with FDR control (Benjamini–Hochberg) across all tested associations. Cross marks (×) denote associations that remain significant after FDR control (FDR<0.05). Smoking was modeled as current or former vs non-smoker; alcohol intake frequency was modeled as an ordinal UK Biobank variable (per category). KDM = Klemera–Doubal Method; PhenoAge = Phenotypic Age; ProtAge = proteomic aging clock; MetaboAge = metabolomic aging clock; HD = homeostatic dysregulation; FI = frailty index; TL = telomere length; PRS = polygenic risk score; BMI = body mass index; FDR: Benjamini–Hochberg false discovery rate (q-value).

***Figure A.5.* ROC and precision–recall curves for stepwise ADRD prediction models in the held-out test set.** Receiver operating characteristic (ROC) curves (Panel A) and precision–recall (PR) curves (Panel B) are shown for the six stepwise XGBoost models evaluated on the held-out test set from a stratified 70/30 train–test split. Models correspond to Table 2: Base (age + sex), Model 1 (+ lifestyle: smoking, alcohol intake frequency, body mass index, education), Model 2 (+ PRS including *APOE*), Model 3 (+ BA: PhenoAge, frailty index, telomere length), Model 4 (+ ProtAge), and Model 5 (+ MetaboAge). The diagonal dashed line in Panel A indicates no-discrimination performance. In Panel B, the dashed horizontal line indicates the no-skill baseline equal to the ADRD event prevalence in the test set. Curves illustrate that ROC AUC plateaus after inclusion of biological aging measures, whereas PR performance continues to improve with addition of omics clocks under class imbalance. ADRD: Alzheimer’s disease and related dementias; PRS: polygenic risk score; PhenoAge = Phenotypic Age; ProtAge = proteomic aging clock; MetaboAge = metabolomic aging clock.

***Figure A.6.* Calibration of the full prediction model on the held-out test set.** Calibration plot comparing mean predicted ADRD risk to the observed event rate across deciles of predicted risk in the 30% test split. Points indicate decile-level mean predicted risk and observed event rate; vertical bars represent approximate 95% binomial confidence intervals for the observed event rate. The dashed diagonal line indicates perfect calibration (observed = predicted), and the solid line shows calibration based on logistic recalibration of the predicted probabilities. The model demonstrated good calibration (slope = 1.05; intercept = 0.04) with a Brier score of 0.0209. Model 5: the final integrated model includes all predictors (Table 2); ADRD: Alzheimer’s disease and related dementias.

**Figure A.7.** **Decision curve analysis for the final integrated ADRD prediction model (UK Biobank held-out test set)**. Net benefit is shown across threshold probabilities from 0 to 0.10. “Treat none” corresponds to no individuals being classified high risk, and “Treat all” corresponds to classifying all individuals as high risk. A higher net benefit indicates better clinical decision utility at that threshold probability. The threshold range focuses on low predicted risks expected under low ADRD incidence.

***Figure A.8.* SHAP summary plot for predictors in the fully integrated model (Model 5).** SHAP (SHapley Additive exPlanations) values were computed in the held-out UK Biobank test set for the final integrated XGBoost model (Model 5). Each dot represents one participant for one predictor. The x-axis shows the SHAP value, where positive values indicate that the predictor increased the model-predicted ADRD risk for that participant and negative values indicate that the predictor decreased predicted risk. Predictors are ordered by mean absolute SHAP value, representing their average contribution to model predictions across the held-out test set. Dot color denotes the observed feature value, with higher values shown in red and lower values shown in blue. PRS and chronological age showed the largest average contributions, followed by ProtAge and PhenoAge. MetaboAge contributed more modestly and showed a model-conditional pattern in which lower MetaboAge values tended to be associated with higher predicted risk, likely reflecting shared variance among correlated aging measures. Alcohol intake frequency reflects self-reported frequency of alcohol intake; smoking is coded as current/former vs non-smoker; low education is defined as <10 years of formal education; men indicates sex coded as male. ADRD: Alzheimer’s disease and related dementias; SHAP: SHapley Additive Explanations; PRS: polygenic risk score; CA: chronological age; ProtAge: proteomic aging clock; MetaboAge: metabolomic aging clock; PhenoAge: phenotypic age; FI: frailty index; TL: telomere length; BMI: body mass index.

***Figure A.9.* Sensitivity analyses of the integrated multivariable model (Model 5) across subgroups and modeling assumptions.** Forest plot comparing AUC values (with 95% confidence intervals) for ADRD prediction under seven scenarios: handling of missing data (complete-case vs. imputation), prediction horizons (5-year vs. 9-year), lifestyle covariate adjustment (with vs. without), *APOE* inclusion in PRS, *APOE-ε4* carrier status (ε4 carriers: genotypes ε2/ε4, ε3/ε4, ε4/ε4; non-carriers: ε2/ε2, ε2/ε3, ε3/ε3), sex-stratified performance, and age-at-onset stratification (early vs. late). ADRD: Alzheimer’s disease and related dementias; AUC: area under the Receiver Operating Characteristic (ROC) curve; PRS: polygenic risk score; *APOE*: apolipoprotein E.

***Figure A.10.*** **Sensitivity analysis for Figure 5 excluding early events (≤100 days)**. Fine–Gray CIF curves for ADRD stratified by predicted 5-year risk group (top 25% vs bottom 75%) after excluding participants with very early events (as defined in the sensitivity analysis). Death was treated as a competing event. Shaded areas indicate 95% confidence intervals. The sHR and *P*-value compare the highest-risk vs lower-risk groups. ADRD: Alzheimer’s disease and related dementias; sHR: subdistribution hazard ratio.

***Figure A.11.* External validation of the full ADRD prediction model in TwinGene: ROC and precision–recall curves**. Receiver operating characteristic curve and precision–recall curve for the covariate-augmented full TwinGene validation model using 5-fold out-of-fold predicted risks. The model was evaluated in the complete-case TwinGene downstream validation cohort using harmonized available predictors corresponding to the UK Biobank integrated prediction framework. The full out-of-fold model achieved ROC-AUC = 0.757 (95% CI: 0.706–0.794) and AUPRC = 0.223 (95% CI: 0.171–0.265). The dashed horizontal line in the precision–recall plot indicates the no-skill baseline corresponding to ADRD prevalence in TwinGene (8.8%). Table A.11 summarizes complementary repeated-split performance stability across the stepwise predictor sequence. ADRD = Alzheimer’s disease and related dementias; ROC-AUC = area under the receiver operating characteristic curve; AUPRC = area under the precision–recall curve.

***Figure A.12.* Cumulative incidence of ADRD by predicted-risk group in TwinGene.** Fine–Gray cumulative incidence curves for incident ADRD in TwinGene stratified by 5-fold out-of-fold predicted risk from the full TwinGene validation model. Participants were classified into the top 25% predicted-risk group versus the lower 75% predicted-risk group. Death before ADRD was treated as a competing event. The top 25% predicted-risk group showed substantially higher cumulative incidence of ADRD than the lower 75% group (subdistribution hazard ratio [sHR] = 4.02, 95% CI: 3.25–5.02, *P* < 0.001). ADRD = Alzheimer’s disease and related dementias; sHR = subdistribution hazard ratio.

**Supplementary Methods: Sensitivity Analyses and Robustness Checks (Methods A.M1)**

**Overview and general modeling conventions**

All sensitivity analyses were designed to assess the robustness of ADRD prediction and effect estimates to (i) subgroup definitions, (ii) PRS specification, (iii) feature set composition, (iv) follow-up horizon and missing-data handling, and (v) time-scale choice in survival modeling. Medication use, hypertension, diabetes, and cardiovascular disease were not included as separate sensitivity analyses. These variables are relevant to ADRD risk but may partly overlap with, mediate, or be downstream of systemic biological-aging measures. Medication variables also require careful harmonization of indication, duration, adherence, and disease severity. We therefore treated these as important future extensions rather than additional adjustment variables in the primary multi-omics aging prediction framework. Unless otherwise stated, analyses used the same ADRD outcome definition as the primary analysis and were performed on the same held-out test set (for predictive performance) or on the full eligible analysis set (for survival models). Continuous predictors were standardized to z-scores (per SD) when estimating hazard ratios/subdistribution hazard ratios to ensure comparability across measures.

Train–test split and leakage control (applies to all ML sensitivity analyses). Primary analyses used a reproducible stratified 70/30 split (as in the main analysis); repeated-split stability analyses used 100 repeated stratified 70/30 splits as described below. All preprocessing steps requiring fitted parameters (e.g., imputation models, transformations, and omics-clock training) were performed using the training split only, then applied to the held-out test split.

Performance metrics and uncertainty. Discrimination was summarized using area under the ROC curve (AUC) and area under the precision-recall curve (AUPRC) on the held-out test set. 95% CIs for AUC were obtained using DeLong’s method, and 95% CIs for AUPRC were obtained via stratified nonparametric bootstrap resampling of the test set (e.g., 2,000 resamples; percentile-based CIs, stratified by ADRD status). Pairwise AUC comparisons were conducted using DeLong’s test on the same test set. PR and ROC curves were produced from the test-set predicted probabilities for each model. Given the low outcome prevalence, PR curves were emphasized as a complementary view of performance under class imbalance.

Threshold-based classification summaries. Because AUC/AUPRC summarize performance across all thresholds, we additionally reported operating-point performance at a pre-specified probability threshold. Confusion matrices (2×2 tables) were computed for the fully integrated model (Model 5) at risk-enrichment thresholds corresponding to the top 5% and top 10% of predicted risk in the held-out test set, as well as at an illustrative default probability threshold of 0.5. These thresholds were used to summarize TP, FP, FN, TN, sensitivity, specificity, PPV, NPV, F1, and accuracy. The 0.5 threshold is reported only as a descriptive operating point and was not optimized for this low-incidence outcome.

For horizon-specific discrimination analyses, the binary outcome was redefined as incident ADRD within the specified horizon using time-to-event and censoring rules consistent with the primary pipeline.

**Repeated-split stability analysis and benchmarking**

To assess robustness to split variability in this low-incidence outcome, we repeated the full prediction pipeline across 100 reproducible stratified 70/30 train–test splits, stratified by incident ADRD status, sex, and baseline age bin (5-year bins). Each repeat used a deterministic replicate-specific seed derived from a fixed master seed. Within each repeat, all preprocessing steps requiring fitted parameters (imputation, transformations, outlier handling, and omics-clock derivation where applicable) were performed in the training split only and applied to the held-out test split; discrimination metrics (ROC-AUC and AUPRC) were computed once on the held-out test split. We summarize XGBoost stability as the mean and empirical 2.5th–97.5th percentiles across repeats (***Table A.5***). In parallel, we benchmarked against a simpler conventional model (logistic regression) fit on the same training splits and evaluated on the corresponding test splits using the Base (age + sex) and Full (Model 5 predictor set) specifications, summarized similarly across repeats (***Table A.6***). These repeated-split summaries quantify robustness to resampling variability and are distinct from within-split confidence intervals reported above.

**End-to-end analytic pipeline**

To clarify how the modeling components relate, the end-to-end pipeline followed a fixed data flow: (1) define the analytic cohort and perform a reproducible, stratified 70/30 train–test split; (2) derive ProtAge and MetaboAge within the training split only using cross-fitting, then generate clock predictors in the held-out test split using the trained clock models; (3) fit stepwise XGBoost prediction models in the training split and evaluate discrimination and calibration once on the fixed held-out test split; (4) fit Fine–Gray competing-risk regression in the training split using the final integrated predictor set (Model 5) to estimate absolute ADRD risk at prespecified horizons with death as a competing event; and (5) assess clinical interpretability via risk stratification (e.g., top-risk quantiles) and cumulative incidence curves in UKB and external validation (TwinGene).

**A. Prediction sensitivity analyses (XGBoost models)**

**A.1 Stratified performance by age at onset (late-onset vs early-onset)**

To assess generalizability across onset-age strata, model discrimination was recalculated separately in late-onset ADRD and early-onset ADRD strata. Early-onset ADRD was defined as age at ADRD diagnosis <65 years and late-onset as ≥65 years. Early-onset ADRD was rare (e.g., N ≈ 21), and therefore discrimination estimates in that subgroup were expected to be unstable and interpreted cautiously.

**A.2 Sex-stratified performance**

We repeated evaluation stratified by sex (women vs men) using the same trained modeling pipeline and computed test-set AUC within each stratum. CIs were computed using stratified bootstrap within each sex stratum.

**A.3 PRS specification: including vs excluding the *APOE* locus**

To quantify the dependence of model performance on *APOE*, we repeated model evaluation using a PRS with *APOE* included (primary) and a PRS excluding the *APOE* locus (sensitivity). This isolates the contribution of non-*APOE* polygenic signal. All other predictors and modeling steps were unchanged, and the same held-out test set was used for comparability.

**A.4 *APOE-ε4* status stratification**

We additionally evaluated discrimination separately among *APOE-ε4* carriers and non-carriers. These stratified evaluations used the PRS including the *APOE* locus (to reflect the primary PRS definition), and results were interpreted as subgroup performance rather than an independent *APOE*-free genetic evaluation. Because carrier strata can be imbalanced, bootstrap CIs were computed with stratification by ADRD status within each *APOE* group.

**A.5 Feature set sensitivity: removing lifestyle covariates**

To test the incremental contribution of lifestyle/demographic covariates beyond age/sex/PRS/biological aging measures, we refit/evaluated models after removing lifestyle covariates (smoking, alcohol, body mass index, education) and compared AUCs to the full model.

**A.6 Follow-up horizon sensitivity (5-year vs 9-year)**

To examine time-horizon robustness, we evaluated discrimination for predicting ADRD within alternative horizons (e.g., 5-year vs 9-year) by redefining the binary outcome as incident ADRD within the horizon. Participants without an ADRD diagnosis before the horizon were treated as non-ADRD participants for that horizon (with appropriate censoring handling as defined in the primary pipeline). AUC was computed for each horizon using the same model structure.

**A.7 Order dependence of stepwise model building (robustness check)**

Because multiple biological aging predictors are correlated, we assessed whether stepwise gains depended on the order in which predictor blocks were added. Using a fixed held-out test set and fixed modeling protocol, we refit stepwise models under several alternative block-entry orders (e.g., adding PRS earlier vs later; adding omics clocks earlier vs later; adding both omics clocks simultaneously) and compared the resulting AUCs. To ensure comparability across orders, we used a single complete-case dataset across all candidate predictors and constructed one consistent design matrix so that feature columns matched across models. We additionally reported the observed AUC range across orderings to demonstrate stability of final-model discrimination.

**B. Survival-model sensitivity analyses**

**B.1 Fine–Gray competing-risk modeling (primary survival framework)**

We modeled incident ADRD with death as a competing event using Fine–Gray subdistribution hazards regression. Time was measured as time since baseline in the primary survival analysis. Individuals were followed from baseline until ADRD diagnosis, death, or administrative censoring.

Competing-risk event coding. We defined a three-level status indicator:

• 0 = censored,

• 1 = ADRD event,

• 2 = death prior to ADRD.

Observed follow-up time was defined as the minimum of the ADRD time, death time, and censoring time.

Time-unit harmonization. Because UKB-derived event times can be stored in different units across variables, all survival times were harmonized to a single unit (days) prior to model fitting. Specifically, ADRD times were treated as days; death time and length of follow-up were converted to days when their distributions indicated they were recorded in years.

Administrative censoring. For competing-risk estimation and cumulative incidence curves, we applied a consistent administrative censoring horizon across participants (end of follow-up), rather than case-specific censoring that could occur earlier than a participant’s recorded event time. This avoids artifacts in cumulative incidence estimation.

**B.2 Sensitivity: age as the time scale (Fine–Gray)**

Because ADRD risk is strongly age-dependent, we performed a sensitivity analysis using age as the time scale, rather than time since baseline. In this approach, each participant entered the risk set at baseline age and exited at baseline age plus observed follow-up time. In this sensitivity model, baseline age was not additionally included as a covariate because age was already represented by the time axis. Results were compared qualitatively to the primary time-since-baseline Fine–Gray model to confirm that inference about key predictors was not driven by the choice of time scale.

**B.3 Univariate survival associations: cause-specific Cox vs Fine–Gray**

To assess robustness of unadjusted associations to competing-risk framework choice, we estimated predictor effects using:

1. Cause-specific Cox proportional hazards models treating death as censoring (event = ADRD; censoring = death or end of follow-up), and
2. Fine–Gray competing-risk models treating death as a competing event.

Each predictor was modeled separately (univariate), scaled per SD, and reported as HR (Cox) or sHR (Fine–Gray) with 95% confidence intervals. This provided a side-by-side comparison of effect magnitudes across frameworks.

**B.4 Early-event exclusion sensitivity**

To test whether early events drove risk separation, we repeated the competing-risk analysis after excluding individuals with very early ADRD events (≤100 days from baseline). We compared cumulative incidence curves and subdistribution hazard ratios to the primary analysis.

**B.5 Competing-risk framework sensitivity**

Fine–Gray vs cause-specific Cox (multivariable). As an additional robustness check, we fit multivariable cause-specific Cox models using the same covariate set as the Fine–Gray models and compared direction/magnitude of key predictors (noting that HR and sHR are not numerically identical but should be directionally consistent when competing risks do not dominate).

**C. Missing-data handling and imputation**

**C.1 Missing data and Multiple Imputation by Chained Equations (MICE)**

Missing values were handled using two complementary approaches to assess robustness: complete-case analysis, which was the primary analytic approach, and multiple imputation as a sensitivity analysis. Comparing complete-case and imputed analyses allowed us to assess whether final model performance was robust to alternative missing-data handling assumptions and to evaluate model behavior in a broader UK Biobank biomarker source population.

The primary complete-case analysis included 16,215 UK Biobank participants, including 397 incident ADRD cases and 15,818 non-cases. For the imputation sensitivity analysis, we used a broader imputation-eligible UK Biobank biomarker source sample with available ADRD outcome status, biological-aging marker information, and follow-up data. This imputation-eligible sample included 40,145 participants, comprising 843 incident ADRD cases and 39,302 non-cases. Among these participants, 37,726 had at least one missing predictor or covariate value imputed. Thus, the imputation sensitivity analysis evaluated the final prediction framework in a larger sample than the primary complete-case analysis while retaining the same ADRD outcome definition and train/test evaluation structure.

For the imputation sensitivity analysis, we used Multiple Imputation by Chained Equations (MICE) and generated 10 imputed datasets. The imputation model included the main prediction variables: chronological age, sex, body mass index, smoking status, alcohol intake frequency, education, the top 10 genetic principal components, ADRD PRS including the *APOE* locus, PhenoAge, frailty index, telomere length, ProtAge, and MetaboAge. *APOE-ε4* carrier status, KDM, and HD were included in sensitivity-specific imputation models when required. ADRD case status and follow-up information were retained for sample definition and final performance evaluation, but were not used as predictors for imputing held-out test-set predictor values.

Variables were imputed according to type: predictive mean matching for continuous variables, logistic regression for binary variables, polytomous regression for categorical variables with more than two levels, and proportional-odds ordinal logistic regression for ordinal variables when appropriate.

To minimize information leakage, the train–test split was defined before imputation. Imputation parameters were estimated using the training split only; held-out test-set rows were imputed without contributing to imputation-model estimation. Model fitting and hyperparameter tuning were performed only in the imputed training datasets, and performance was evaluated only in the corresponding held-out test datasets.

Across the 10 imputed datasets, mean held-out ROC-AUC was 0.879, with values ranging from 0.853 to 0.911, compared with ROC-AUC = 0.899 in the primary complete-case analysis. Mean AUPRC was 0.201, with values ranging from 0.157 to 0.278. MICE convergence was assessed by visual inspection of trace plots across iterations.

**Methods A.M2. External validation and replication in TwinGene**

**Cohort and outcome definition:**

TwinGene was used as an independent Swedish population-based cohort to evaluate external transportability and replication of the UK Biobank integrated ADRD prediction framework. The downstream TwinGene replication dataset was constructed by merging phenotype, genetic, biological aging, proteomic aging, metabolomic aging, lifestyle, and follow-up data. The primary complete-case TwinGene downstream replication cohort included 3,772 participants, with a mean baseline age of 64.2 years, 47.0% men, and a median follow-up of 16.7 years. During follow-up, 331 participants developed incident ADRD and 841 experienced death without ADRD as a competing event. Incident ADRD was ascertained using registry-based diagnoses. Follow-up time was defined from baseline assessment to the earliest of ADRD diagnosis, death, or censoring.

**Predictor set used for TwinGene downstream validation:**

The TwinGene full model was designed to mirror the UK Biobank Model 5 predictor structure as closely as possible using harmonized available predictors. The final TwinGene downstream model included chronological age at baseline, sex, body mass index, smoking status, alcohol intake frequency, education, the first 10 genetic principal components, ADRD polygenic risk score, clinical biological aging measures (PhenoAge, frailty index, and telomere length), proteomic aging, and metabolomic aging. Sensitivity variables included an *APOE*-excluded PRS, the raw UKB-portable MetaboAge prediction before recalibration, and additional clinical biological aging measures (KDM and HD) for correlation or sensitivity analyses.

**ADRD polygenic risk score in TwinGene:**

The ADRD PRS in TwinGene was constructed using the selected ADRD-associated SNP panel and externally derived effect-size weights from the Jansen et al. summary statistics. SNPs were harmonized to the effect allele, and weighted allele dosages were summed across available variants. The primary TwinGene PRS variable was standardized to a z-score within TwinGene before downstream modeling. *APOE* genotype was not entered as a separate predictor in the primary downstream model; *APOE* information was incorporated through the PRS including the *APOE* locus. As a sensitivity predictor, we also retained an *APOE*-excluded PRS, constructed after excluding *APOE*-region variants. In TwinGene, 73 selected SNPs were available and harmonizable after allele alignment; these were used to calculate the primary TwinGene PRS variable.

**Biological aging measures in TwinGene:**

Clinical biological aging measures in TwinGene were derived and harmonized to mirror the UK Biobank framework as closely as possible. We used the same conceptual set of clinical biological aging domains as in UKB: a phenotypic-age measure, a deficit-accumulation frailty measure, and leukocyte telomere length. Specifically, PhenoAge was calculated from available clinical biomarker and chronological age information using the same phenotypic-age framework applied in UKB, subject to biomarker availability in TwinGene. Frailty index was calculated using a Rockwood deficit-accumulation approach, representing the proportion of available health deficits present for each participant. Telomere represented harmonized leukocyte telomere length and was included as the telomere-aging domain. These three variables—PhenoAge, frailty index, and telomere length —were used as the primary clinical biological aging predictors in the TwinGene full model to match the UKB Model 5 predictor structure.

KDM and HD were also available or derivable in TwinGene and were retained for correlation and sensitivity analyses, but they were not included in the primary TwinGene full downstream model. This mirrored the UKB modeling strategy, where PhenoAge, FI, and TL were prioritized to reduce redundancy and collinearity while preserving complementary clinical biological aging domains. Continuous biological aging measures were standardized within TwinGene before downstream prediction and competing-risk analyses.

**TwinGene ProtAge derivation:**

For proteomic aging, we used TwinGene Olink data to replicate the ProtAge algorithm/workflow within TwinGene rather than applying frozen UKB ProtAge coefficients. This was necessary because the Olink feature space and available proteins differed between cohorts. The TwinGene Olink ProtAge input included 6,283 participants with 351 Olink protein measurements. Olink values were used directly, without additional log transformation. The protein feature set was filtered to remove all-missing, constant, near-zero variance, and highly correlated proteins. Missing protein values were imputed using k-nearest-neighbor imputation (k = 9). Outliers were identified using Isolation Forest, with the highest anomaly-score observations excluded/flagged according to the predefined pipeline. ProtAge was then trained to predict chronological age using a sex-stratified stacked ensemble approach. Base learners included XGBoost and LightGBM, with CatBoost used when available; Elastic-Net regression served as the meta-learner. Within each sex stratum, a 70/30 train–test split and 5-fold cross-validation within the training data were used for model development. In held-out TwinGene ProtAge testing, the stacked model achieved correlation = 0.848 with chronological age, RMSE = 4.14 years, and MAE = 3.25 years.

**TwinGene MetaboAge derivation:**

For metabolomic aging, we applied the UKB-portable MetaboAge prediction workflow to TwinGene targeted metabolomics data. The raw TwinGene MetaboAge prediction was retained as MetaboAge_UKBportable_raw_for_calibration. Because of cross-cohort/platform differences in metabolomics distributions, the raw prediction was recalibrated within TwinGene using sex-specific linear recalibration against chronological age. The recalibrated variable was used as the primary MetaboAge predictor in the downstream TwinGene ADRD model. Raw and recalibrated age-acceleration variables were also retained for sensitivity and diagnostic analyses. In held-out TwinGene MetaboAge testing, the stacked model achieved correlation = 0.382 with chronological age, RMSE = 7.43 years, and MAE = 5.90 years.

**Covariate harmonization:**

Covariates were harmonized to correspond as closely as possible to the UKB model. Genetic ancestry adjustment used PC1–PC10. Continuous predictors were standardized before modeling when appropriate, and categorical predictors were encoded consistently with the downstream prediction pipeline.

**TwinGene validation analysis:**

The TwinGene validation analysis evaluated whether the integrated predictor framework retained predictive signal in an independent cohort. Discrimination was assessed using ROC-AUC and AUPRC for incident ADRD prediction. For risk stratification, predicted-risk groups were constructed from the TwinGene downstream model and compared using Fine–Gray competing-risk regression with death before ADRD treated as a competing event. Because the proteomic and metabolomic aging predictors required cohort-specific replication/recalibration, TwinGene analyses are interpreted as external transportability and methodological replication of the integrated framework rather than strict frozen-coefficient validation of every UKB-derived omics component.
